# New Insights Into *Acidithiobacillus thiooxidans* Sulfur Metabolism Through Coupled Gene Expression, Solution Chemistry, Microscopy, and Spectroscopy Analyses

**DOI:** 10.3389/fmicb.2020.00411

**Published:** 2020-03-13

**Authors:** David Camacho, Rodolfo Frazao, Aurélien Fouillen, Antonio Nanci, B. Franz Lang, Simon C. Apte, Christian Baron, Lesley A. Warren

**Affiliations:** ^1^School of Geography and Earth Science, Faculty of Science, McMaster University, Hamilton, ON, Canada; ^2^Department of Biochemistry and Molecular Medicine, Faculty of Medicine, Université de Montréal, Montreal, QC, Canada; ^3^Laboratory for the Study of Calcified Tissues and Biomaterials, Faculty of Dentistry, Université de Montréal, Montreal, QC, Canada; ^4^CSIRO, Land and Water, Lucas Heights, NSW, Australia; ^5^Department of Civil and Mineral Engineering, Faculty of Applied Science and Engineering, University of Toronto, Toronto, ON, Canada

**Keywords:** sulfur metabolism, gene expression, geochemistry, *Acidithiobacillus thiooxidans*, sulfur oxidation, modeling

## Abstract

Here, we experimentally expand understanding of the reactions and enzymes involved in *Acidithiobacillus thiooxidans* ATCC 19377 S^0^ and ${\text{S}}_{2}\,{\text{O}}_{3}^{2-}$ metabolism by developing models that integrate gene expression analyzed by RNA-Seq, solution sulfur speciation, electron microscopy and spectroscopy. The *A. thiooxidans* ${\text{S}}_{2}\,{\text{O}}_{3}^{2-}$ metabolism model involves the conversion of ${\text{S}}_{2}\,{\text{O}}_{3}^{2-}$ to ${\text{SO}}_{4}^{2-}$, S^0^ and ${\text{S}}_{4}\,{\text{O}}_{6}^{2-},$ mediated by the sulfur oxidase complex (Sox), tetrathionate hydrolase (TetH), sulfide quinone reductase (Sqr), and heterodisulfate reductase (Hdr) proteins. These same proteins, with the addition of rhodanese (Rhd), were identified to convert S^0^ to ${\text{SO}}_{3}^{2-}$, ${\text{S}}_{2}\,{\text{O}}_{3}^{2-}$ and polythionates in the *A. thiooxidans* S^0^ metabolism model. Our combined results shed light onto the important role specifically of TetH in ${\text{S}}_{2}\,{\text{O}}_{3}^{2-}$ metabolism. Also, we show that activity of Hdr proteins rather than Sdo are likely associated with S^0^ oxidation. Finally, our data suggest that formation of intracellular ${\text{S}}_{2}\,{\text{O}}_{3}^{2-}$ is a critical step in S^0^ metabolism, and that recycling of internally generated ${\text{SO}}_{3}^{2-}$ occurs, through comproportionating reactions that result in ${\text{S}}_{2}\,{\text{O}}_{3}^{2-}$. Electron microscopy and spectroscopy confirmed intracellular production and storage of S^0^ during growth on both S^0^ and ${\text{S}}_{2}\,{\text{O}}_{3}^{2-}$ substrates.

## Introduction

The stepwise oxidation of reduced sulfur species from sulfide to sulfate can occur via several pathways involving a variety of sulfur oxidation intermediate (SOI) compounds that are dynamically influenced by environmental and geochemical characteristics as well as the microbes involved (Schippers et al., 1996; Schippers and Sand, 1999; Nordstrom, 2015). This range of sulfur oxidation states contributes to a complex, and only partially constrained biogeochemical cycle in which sulfur compounds can be variably reduced, oxidized and disproportionated via abiotic and/or biotic processes depending on environmental conditions (Johnston and McAmish, 1973; Kelly and Baker, 1990; Pronk et al., 1990; Druschel, 2002; Zopfi et al., 2004; Bernier and Warren, 2007; Boyd and Druschel, 2013). The geochemical challenges to closing the sulfur biogeochemical cycle reflect the existence of multiple semi-stable SOI compounds, which are either not comprehensively constrained to date and/or lack readily available analytical methods for their characterization (Miranda-Trevino et al., 2013). For instance, the challenges in measuring polythionates and other higher oxidation state sulfur compounds have impeded the delineation of their roles in the chain of reactions culminating in sulfate (Johnson and Hallberg, 2003; Nordstrom et al., 2015). The complexities of sulfur chemistry underscore the need for mass balance of all sulfur within systems, in order to quantify how much sulfur may be tied up in a currently unidentified or, as referred to here, “other SOI” pool. However, sulfur mass balance is rarely employed in studies of sulfur cycling.

Further, microbial catalysis, dependent on the specific bacteria, growth stage and sulfur substrates involved, is important for initiating or accelerating rates for some of these sulfur oxidation reactions (Bacelar-Nicolau and Johnson, 1999; Druschel et al., 2004; Bernier and Warren, 2005, 2007; Beller et al., 2006; Warren et al., 2008; Bobadilla Fazzini et al., 2013). Several studies have demonstrated flexibility of the sulfur oxidation metabolism by assessing the solution chemical changes in some intermediate sulfur species, or inferred pathways from what is known about identified sulfur metabolism genes within an organism or community (Bobadilla Fazzini et al., 2013; Jones et al., 2014; Yin et al., 2014; Houghton et al., 2016). Intermediate species of sulfur, especially S^0^, ${\text{S}}_{2}\,{\text{O}}_{3}^{2-}$, and polythionates [${\text{S}}_{\text{n}}\,{\text{O}}_{6}^{2-}$ (*n* > 2)], are important in microbial processing of sulfur, even though their concentrations in solution may be low. Indeed, these intermediate sulfur compounds are thought to be involved in the so-called “cryptic” sulfur cycle, an enigmatic process in which sulfur is recycled amongst lower state sulfur species that is not well-characterized to date (Thamdrup et al., 1994; Jørgensen and Nelson, 2004; Canfield et al., 2010; Houghton et al., 2016).

Further, gaps in understanding of which proteins catalyze specific sulfur pathways also exist (Friedrich et al., 2001; Sauvé et al., 2007; Valdes et al., 2011; Jones et al., 2014). The literature to date indicates that some sulfur metabolic enzymes catalyze a broad suite of sulfur oxidative reactions, e.g., the Sox (sulfur oxidizing) complex, while others seem to catalyze more specific sulfur reactions, e.g., Sdo (sulfur dioxygenase) (Kelly et al., 1997; Friedrich et al., 2001; Rohwerder and Sand, 2003; Hensen et al., 2006; Sauvé et al., 2007; Wang et al., 2019). Some microorganisms capable of sulfur oxidation can possess a suite of these genes, enabling them to carry out many different reactions, while others have a more limited set of sulfur genes, restricting them to select reactions only (Hallberg and Johnson, 2003; Ghosh and Dam, 2009; Zhu et al., 2012; Nuñez et al., 2017). Recent works reviewing *Acidithiobacillus* spp. sulfur metabolism have identified diverse pathways for this genus dependent on the species, as well as the sulfur substrate(s) (S^0^, ${\text{S}}_{2}\,{\text{O}}_{3}^{2-}$, ${\text{S}}_{4}\,{\text{O}}_{6}^{2-}$) and the different sulfur metabolism genes available to them (Wang et al., 2019; Zhan et al., 2019). These studies have provided updated models for *A. caldus* and *A. ferrooxidans* based on the existing literature of studies using either genomics, proteomics or sulfur chemistry analyses. For both species, S^0^ metabolism is proposed as oxidation to ${\text{SO}}_{3}^{2-}$ via Sdo, followed by oxidation to ${\text{SO}}_{4}^{2-}$ via the sulfate adenylyltransferase dissimilatory-type (SAT) gene (Wang et al., 2019). While the ${\text{S}}_{2}\,{\text{O}}_{3}^{2-}$ metabolism is proposed to differ between the two species, where in *A. caldus* it is through the S_4_I pathway and Sox complex, and in *A. ferrooxidans* via the S_4_I pathway and thiosulfate dehydrogenase (TSD) (Wang et al., 2019; Zhan et al., 2019). The S_4_I pathway utilizing the *doxD* (thiosulfate:quinone oxidoreductase) and *tetH* (tetrathionate hydrolase) genes (Wang et al., 2019). While further notable genes present in the sulfur metabolism for *Acidithiobacillus* spp. include the *sqr* (sulfide quinone reductase), *sor* (sulfur oxygenase reductase), *rhd* (rhodanese) and the heterodisulfide reductase or Hdr-like complex (*hdrA*, *hdrB*, and *hdrC*) (Ghosh and Dam, 2009; Valdes et al., 2011; Jones et al., 2014; Yin et al., 2014; Wang et al., 2019).

**FIGURE 1:**
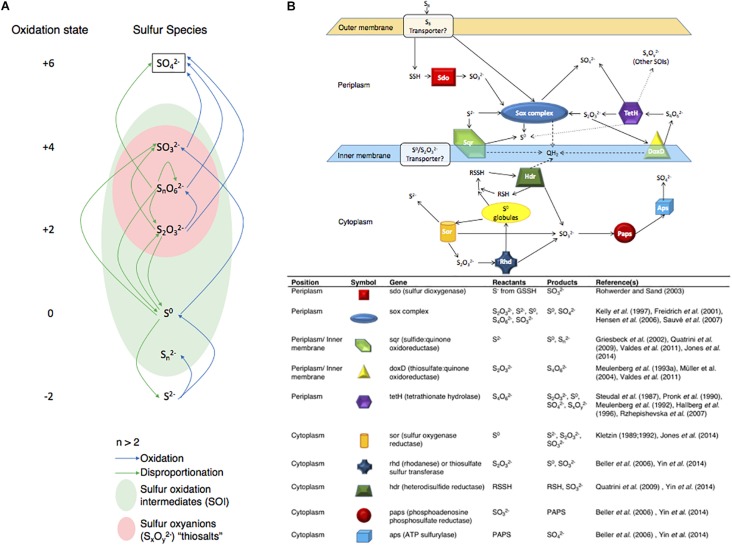
Biological sulfur capabilities and gene layout for *Acidithiobacillus thiooxidans.* **(A)** Biological sulfur reaction capabilities across all known sulfur species and oxidation states and **(B)** a theoretical map for the locations of sulfur genes analyzed and information on potential reactants and products of these genes. Showing proven sulfur reactions in solid arrows, theorized reactions in dotted arrows and cytochrome C proton reactions in dashed arrows. Based on the referenced literature.

Here, the objectives were to characterize both the levels of gene expression at high resolution (RNA-Seq) for *Acidithiobacillus thiooxidans*, and the changes in sulfur speciation associated with its experimental growth on either S^0^ or ${\text{S}}_{2}\,{\text{O}}_{3}^{2-}$ to generate models for *A. thiooxidans* sulfur metabolism. *A. thiooxidans* is a strict autotroph only able to carry out sulfur oxidation/disproportionation reactions (Figure 1A) and a well-studied sulfur oxidizing microorganism (Kelly et al., 1997; Suzuki et al., 1999; Masau et al., 2001; Rohwerder and Sand, 2003). The model organism *A. thiooxidans* ATCC 19377 used here, encodes at least 10 known proteins or protein complexes thought to be involved in sulfur metabolism, which includes the aforementioned S_4_I pathway and Sox complex in the periplasm, and the Hdr-like complex in the cytoplasm (Valdes et al., 2011; Bobadilla Fazzini et al., 2013; Yin et al., 2014) (Figure 1B). Our integrated approach provides important novel insights since previous studies have designed models for this species based solely on solution chemistry (Bobadilla Fazzini et al., 2013) or gene expression (Yin et al., 2014).

## Materials and Methods

### Experimental Design, Cell Growth, and Counting

#### Experimental Design

In order to jointly assess both gene expression and changes in sulfur speciation, the experimental design included collection of samples for cell counts, gene expression, microscopy, S speciation and pH for *A. thiooxidans* grown in both S^0^ and ${\text{S}}_{2}\,{\text{O}}_{3}^{2-}$ treatments over 12 days to ensure both exponential and stationary phases were encompassed in the characterization. Greater details on collection and analyses of samples for each of these variables are provided subsequently.

#### Culture Conditions

*Acidithiobacillus thiooxidans* ATCC 19377 cells were grown in liquid elemental sulfur or thiosulfate media (Staley et al., 1989). The media contained two components, the salt medium and the sulfur source. Elemental sulfur salt medium: (NH_4_)_2_SO_4_, 0.2 g; MgSO_4_ × 7 H_2_O, 0.5 g; CaCl_2_ × 2 H_2_O, 0.331 g; KH_2_PO_4_, 3.0 g; FeSO_4_ × 7 H_2_O, 9.15 mg; distilled water, 1,000 ml. The salt medium was sterilized by passing through a 0.22 μm filter. Elemental sulfur powder was heated in an oven at 100°C for 30 min and the cycle was repeated three times. The salt medium was then added to the culture flasks and the final sulfur concentration was 1% (m/v). Thiosulfate medium: salt medium as above and Na_2_S_2_O_3_ was added at 0.2% (m/v), followed by filter sterilization (0.22 μm filter). For both cultures, the total volume of medium corresponded to a fifth of the total volume of the Erlenmeyer flask. All cultures were initially inoculated at 5% v/v with cultures pre-grown in the corresponding media; the inoculant bacteria were washed with sterile 1% NaCl solution prior to inoculation. All cultures were grown under aerobic conditions at 30°C and flasks were shaken at 120 rpm.

#### Fluorescence-Activated Cell Sorting (FACS)

Cells were harvested at the desired time points (days 1, 2, 3, 4, 5, 8, 10, and 12) and washed with 1% NaCl. Optical density (O.D.) values were determined to generate cell counts; however errors introduced by S^0^ clumping precluded their use for these experiments. Thus, for the growth curves, 2 μl of the Live/Dead marker mixture of component A and component B at a ratio of 1:1 (L7012 LIVE/DEAD^®^ BacLight, Bacterial Viability Kit, Thermo Fisher Scientific) were added to 1.5 ml of bacterial suspension. The rationale behind the Live/Dead stain is that all cells will be stained green, because SYTO 9 penetrates into live and dead cells and stains their DNA, whereas propidium iodide (red stain) penetrates only into dead or damaged cells with leaky membranes staining their DNA. For the negative control (dead cells), the cells were first washed with 1% NaCl and then incubated in 70% ethanol for 1 h, followed by washing with 1% NaCl. Propidium iodide (Component B) was added (0.66 μl for 1 ml of bacterial suspension). For the positive control, 0.66 μl of SYTO 9 (Component A) was added to 1 ml of bacterial suspension. All samples were incubated in the dark at room temperature for 15 min, followed by counting in a FACS BD Canto II instrument. Experiments were conducted in triplicates.

### Genetic Methods and Analyses

#### DNA Purification

Genomic DNA was purified from cells from 50 ml bacterial culture grown on elemental sulfur by manual cell disruption with a pestle in the presence of small glass beads (<106 μm diameter; sufficient to form a thick paste). Genomic DNA was purified from combined washes with TE buffer (10 mM Tris, 1 mM EDTA, pH 8) following essentially the instructions of the Qiagen Genomic G20 kit, resulting in 10 μg of purified total DNA.

#### Illumina DNA Sequencing

For paired-end Illumina sequencing (MISEQ-PE300, i.e., 300 nucleotides read length), a TruSeq library was constructed with sized DNA fragments (570 to 650 bp). The reads received from the sequencing service (McGill and Génome Québec Innovation Centre; Montreal, QC, Canada) were cleaned from adapters and quality-clipped with the Trimmomatic software (Bolger et al., 2014), resulting in a total of 2,254,174 read pairs. In addition, a Nextera mate-pair library (insert size 7–8 kbp) was sequenced on two flow cells of Illumina HISEQ (rapid mode; 150 nucleotides read length), and cleaned with Trimmomatic (8,224,769 read pairs).

#### Genome Assembly and Annotation

The genome was assembled with Spades v. 3.6.1 (Bankevich et al., 2012) using a coverage cutoff value of 3.0. The resulting set of contigs was annotated with Prokka v.1.13.3 (Seemann, 2014).

#### Total RNA Extraction

Cells were harvested on day 3 (exponential phase; pH 2.5) and 5 (stationary phase; pH 1.5) for S^0^ media and day 5 (stationary phase; pH 2.5) for ${\text{S}}_{2}\,{\text{O}}_{3}^{2-}$ media, and washed with ice-cold NaCl 1%. They were then lysed and total RNA was extracted using the High Pure RNA Isolation Kit (Roche). Instead of 4 μl of lysozyme as indicated in the kit, 20 μl were added to efficiently break the cells. The lysozyme solution was prepared from egg white lysozyme (Bio Basic, Inc.; activity: 20,000 U/mg) at a final concentration of 50 mg/ml. The genomic DNA was removed using the TURBO DNA-*free* Kit^TM^ (Ambion). The concentration of total RNA was determined using a Nanodrop instrument and the quality of the preparation was assessed by agarose gel electrophoresis to monitor 16S and 23S ribosomal RNA. Samples were conserved at −80°C; experiments were conducted in biological triplicates.

#### High-Throughput RNA Sequencing and Bioinformatics

Sequencing was done using Illumina Hi-seq technology (100 bases paired-end). Quality controls, DNA library construction from isolated RNA and sequencing were performed at the Génome Québec Innovation Centre (Montreal, QC, Canada). Bioinformatics analysis was done using software available on the Galaxy server^1^ (Giardine et al., 2005; Blankenberg et al., 2010; Goecks et al., 2010). Full-length reads (100 bases) were trimmed so that only portion 11 to 80 of each read was conserved. Quality control of the reads was done using FastQC (Galaxy Tool Version 0.63) before and after trimming to ensure quality of the reads. The quality format was changed to “Sanger & Illumina 1.8 +” using FASTQ Groomer (Galaxy Tool Version 1.0.4). Reads were mapped as paired-end using Tophat (Galaxy Tool Version 0.9). The mean inner distance between mate pairs was set to 150 bases and the standard deviation to 20. The reference genome of *A. thiooxidans* (Valdes et al., 2011) was used as guide to help align the reads and the defaults parameters of Tophat were selected. Finally, differential expression was analyzed using Cufflinks (Galaxy Tool Version 2.2.1.0). The “max intron length” was set to 300,000, the “min isoform fraction” was set to 0.1 and the “pre mRNA fraction” to 0.15. Cufflinks only counted fragments compatible with the reference annotation of the genome and it performed a biased correction using the genome assembly. Default Cufflinks parameters were selected.

### Sulfur Chemistry Methods and Analyses

#### Biogeochemical Experiments

Nine sterile 1 L flasks were prepared for batch experimentation: six containing salt medium with 1% S^0^ and three with 0.2% ${\text{S}}_{2}\,{\text{O}}_{3}^{2-}$ culture medium, followed by *A. thiobacillus* inoculation as described above. For each treatment, one flask was sacrificed for sulfur chemical analyses from the S^0^ cultures on days 0, 1, 2, 3, 4, and 5 and from the ${\text{S}}_{2}\,{\text{O}}_{3}^{2-}$ cultures on days 0, 2, and 4. For each sampling time, the bulk solution pH was measured (Denver Instrument Model 225, Bohemia, NY, United States) prior to sampling for sulfur analyses. Triplicate samples were then collected for dissolved (<0.45 μm), total sulfur (ΣS_aq_) and sulfur speciation (${\text{SO}}_{4}^{2-}$, S^2–^, ${\text{S}}_{2}\,{\text{O}}_{3}^{2-}$, S^0^, and ${\text{SO}}_{3}^{2-}$) analyses as described subsequently.

#### ΣS_aq_ – Determination by ICP-AES

For total S (ΣS_aq_), 40 ml of water samples were filtered by Pall Acrodisc^®^ 25 mm 0.45 μm Supor^®^ membrane via polypropylene syringes into 50 ml Falcon^TM^ tubes, followed immediately by addition of 80 μL of HNO_3_ (Optima grade, Fisher Chemical) to each tube before storing at 4°C until analyses. To enable sulfur mass balance calculations, ΣS_aq_ analyses were performed by inductively coupled argon plasma emission spectrometry (ICPAES) (Varian730 ES, Mulgrave, VIC, Australia) using the operating conditions recommended by the manufacturer. Sulfur calibration standards were prepared from certified reference stock solutions (AccuStandard, New Haven, CT, United States) in 2% v/v HNO_3_. The limit of detection (LOD) for sulfur was 1 mg L^–1^ (calculated as three times the standard deviation of the mean blank). Subtracting the sum of all measured solution sulfur species concentrations, described subsequently (${\text{SO}}_{4}^{2-}$, S^2–^, ${\text{S}}_{2}\,{\text{O}}_{3}^{2-}$, S^0^, and ${\text{SO}}_{3}^{2-}$) from the total sulfur (ΣS_aq_) concentration, allowed us to quantify the concentration of S occurring within an unresolved or “Other” SOI pool.

#### ${\text{SO}}_{4}^{2-}$ and S^2–^ – Determination by Spectrophotometry

At each sampling time point, samples were immediately fixed and analyzed using the HACH SulfaVer 4 Method and Methylene Blue Method for ${\text{SO}}_{4}^{2-}$ and S^2–^, respectively (Hach Company, Loveland, CO, United States) by spectrophotometry (Pharmacia Biotech Ultrospec 3000 UV/Visible Spectrophotometer).

#### ${\text{S}}_{2}\,{\text{O}}_{3}^{2-}$, S^0^, and ${\text{SO}}_{3}^{2-}$ – Determination by HPLC

Sampling and analyses for individual SOI species ${\text{S}}_{2}\,{\text{O}}_{3}^{2-}$, S^0^, and ${\text{SO}}_{3}^{2-}$ were concomitant with those for total S, ΣS_aq_, and redox end members, ${\text{SO}}_{4}^{2-}$ and S^2–^. At each sampling time point, samples were taken and immediately preserved using a monobromobimane derivatization procedure for SOI analyses by HPLC (Rethmeier et al., 1997). The Alltima HP C18 (5 μm × 150 mm × 4.6 mm) reverse phase column and Shimadzu LC-20AD prominence HPLC instrument were used for all SOI analyses. Solvents used in protocols were: A = Water, B = Methanol, C = Acetonitrile, D = Acetic acid 0.25% v/v pH 3.5 adjusted with NaOH (1N). ${\text{S}}_{2}\,{\text{O}}_{3}^{2-}$ and ${\text{SO}}_{3}^{2-}$ were assessed via fluorescence excitation at 380 nm and emission at 480 nm. Standards and calibrations for ${\text{S}}_{2}\,{\text{O}}_{3}^{2-}$ (0–10 mM) and ${\text{SO}}_{3}^{2-}$ (0–1.7 mM) were made with ${\text{Na}}_{2}\,{\text{S}}_{2}\,{\text{O}}_{3}^{2-}$ and ${\text{Na}}_{2}\,{\text{SO}}_{3}^{2-}$, respectively. The thiosulfate and sulfite elution protocol was as follows: 0–1 min, 1 ml/min flow; 1–6 min, 1 to 0.85 ml/min flow linear gradient; 0–8 min B 35%, D 65% to B 40%, D 60% linear gradient, oven heated to 35°C. Sample size was 5 μl and elution times were 3 min for ${\text{SO}}_{3}^{2-}$ and 6.5 min for ${\text{S}}_{2}\,{\text{O}}_{3}^{2-}$. S^0^ was extracted with chloroform from both filtered (<0.45 μm, i.e., colloidal) and unfiltered samples (i.e., particulate and/or colloidal) and analyzed with reverse-phase HPLC and UV-absorption at 263 nm. Standards and calibrations (0–32 mM) were made from S^0^ dissolved in chloroform. S^0^ elution protocol: 1 ml/min flow, B 65%, C 35% isocratic; the sample size was 10 μl and the elution time was at 5 min.

### Microscopy and Spectroscopy Analyses

#### Transmission Electron Microscopic (TEM) Analysis

25 ml cultures of bacteria were grown in 1% S^0^ or 0.2% ${\text{S}}_{2}\,{\text{O}}_{3}^{2-}$ media, respectively. Cells were sedimented and rinsed three times with 0.1M phosphate buffer at pH 7.2 to eliminate the remaining medium. Cells were fixed with 4% paraformaldehyde (Acros Organics, Morris Plains, NJ, United States) and 0.1% glutaraldehyde (Electron Microscopy Sciences, Fort Washington, PA, United States) for 30 min at 4°C, followed by three wash with 0.1M phosphate buffer before osmification using 1% osmium tetroxide for 1 h at room temperature. The pellets were dehydrated using a graded ethyl-alcohol series and then processed for embedding in epon (Marivac, Halifax, NS, Canada). Ultrathin sections of 80–100 nm thickness were cut with a diamond knife, collected on Formvar-carbon (polyvinyl formate) coated 200-mesh nickel grids. Sections were then stained with 2% uranyl acetate and lead citrate and examined with a FEI Tecnai 12 (Eindhoven, Netherlands) transmission electron microscope operating at 80 kV.

#### Energy-Dispersive X-Ray Spectroscopy and Wavelength-Dispersive Spectroscopy Analysis

Bacterial sections were imaged using a transmission electron microscope (Jeol JEM-2100F, JEOL, Ltd., Tokyo, Japan) equipped for elemental analysis by energy-dispersive X-ray spectroscopy (EDS). In addition, a scanning electron microscope (Jeol JSM-7600F, JEOL, Ltd., Tokyo, Japan) was used for wavelength dispersive X-ray Spectroscopy (WDS) analysis to obtain a better isolation of the peaks of interest for quantitative analysis.

### Statistical Analyses

Growth curve and pH results for the two treatments were compared by *t*-test analyses: paired two samples for means via Microsoft Excel 2016, with each treatment having three replicates per data point. RNA-seq analysis is a whole genome approach allowing the detection of low and highly expressed genes using the parameter fragments per kilobase of transcript per million mapped reads (FPKM), and the standard deviations between each treatment’s triplicates. Further analyses on FPKM values was carried out to make pairwise comparisons using independent *t*-test on the FPKM between RNA-seq experiments and for the relative levels of gene expression based on Log2 values between samples for the suite of known sulfur genes: (1) across growth curve stage within the S^0^ media, (2) between S^0^ and ${\text{S}}_{2}\,{\text{O}}_{3}^{2-}$ media at the same solution pH and (3) at the same growth curve stage via Microsoft Excel 2016. The chemical relationships between the different S species and [H^+^] (pH) were tested using ANOVA regression statistics via Microsoft Excel 2016 and significance of *p*-value < 0.05 are stated. Intracellular S^0^ globules were analyzed after TEM to determine the quantity and size of globules found inside the cells using Image J software.^2^ Manual modeling and stoichiometric balancing methodology is presented in Supplementary Text.

## Results

### Growth, pH, and Sulfur Species Related to Gene Expression

**FIGURE 2:**
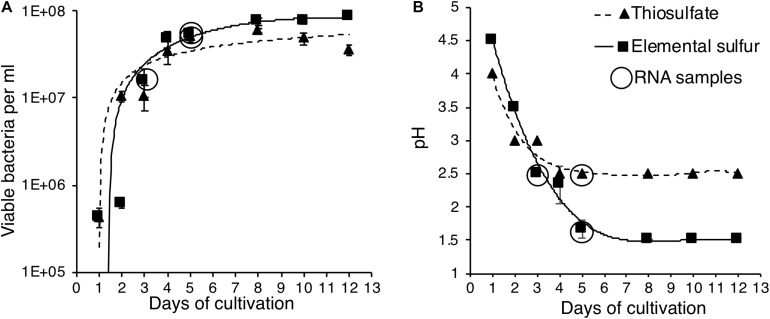
Identification of growth and changes of pH for *A. thiooxidans* grown with alternative energy sources. **(A)** Growth curves for *A. thiooxidans* grown with S^0^ (squares, solid line) and ${\text{S}}_{2}\,{\text{O}}_{3}^{2-}$ (triangles, dotted line) as energy source. **(B)** Changes of pH in the media for *A. thiooxidans* grown with S^0^ (squares, solid line) and ${\text{S}}_{2}\,{\text{O}}_{3}^{2-}$ (triangles, dotted line) as energy source. Time points of samples analyzed by RNA-Seq analyses are indicated by circles. The data are the result of analyses conducted in triplicates and where not visible, error bars for pH measurements were smaller than the symbols plotted for mean pH values.

We cultivated *A. thiooxidans* on minimal media with S^0^ or ${\text{S}}_{2}\,{\text{O}}_{3}^{2-}$ as the source of energy. The results indicate that the organism can extract energy with equal efficiency from both compounds, as evidenced by statistically identical growth patterns for the two media (*p* < 0.05) (Figure 2A). However, the amount of acid generated was higher in the S^0^ media (final pH of 1.5 compared to 2.5 in the ${\text{S}}_{2}\,{\text{O}}_{3}^{2-}$ media) with a corresponding higher slope of pH decrease (0.68 vs. 0.45) as compared to the results on ${\text{S}}_{2}\,{\text{O}}_{3}^{2-}$ media over the experimental time period (days 0–5) (Figure 2B). These results indicate *A. thiooxidans* catalyzes sulfur substrate-dependent metabolic reactions, which may correspondingly be reflected in differential gene induction profiles.

### Genomic Analyses

#### Sequencing, Assembly, and Annotation of the *A. thiooxidans* Genome

**FIGURE 3:**
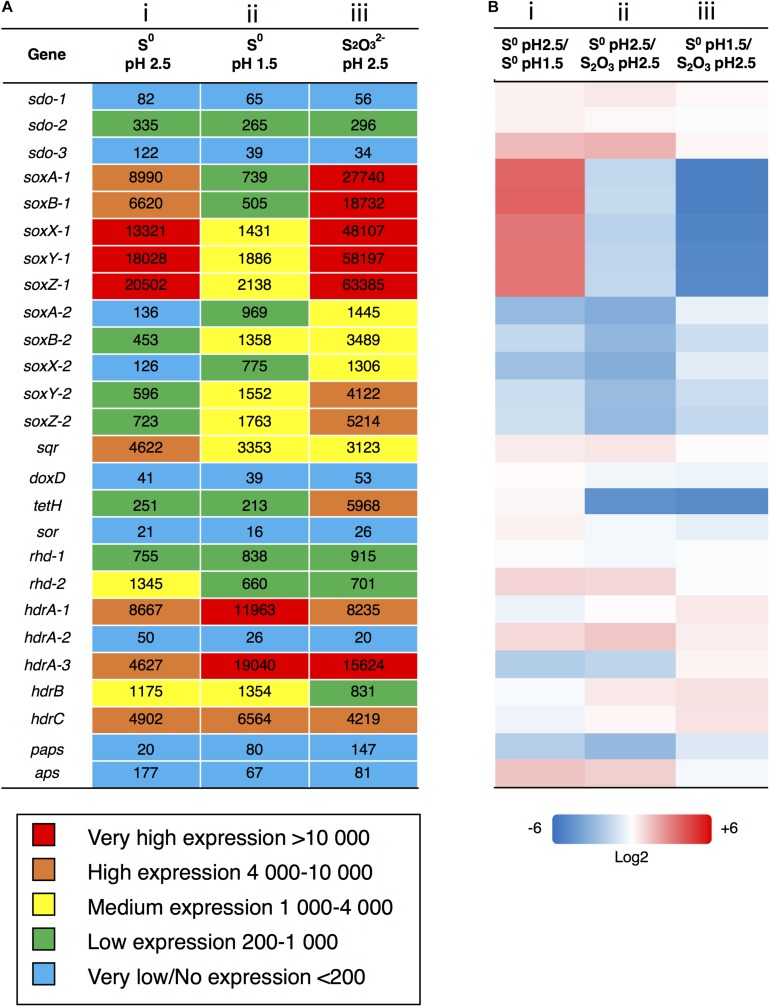
Analysis of gene expression after growth with S^0^ or ${\text{S}}_{2}\,{\text{O}}_{3}^{2-}$ as energy source. **(A)** Gene expression based on FPKM values was analyzed after growth on S^0^ to **(i)** pH-value 2.5 and at **(ii)** 1.5 and on **(iii)** ${\text{S}}_{2}\,{\text{O}}_{3}^{2-}$ to pH-value 2.5. Color scale against indicates relative expression values with blue being the very low, green is low, yellow is intermediate, orange is high and red represents very highly expressed genes. **(B)** Comparative gene expression for FPKM values based on Log2 ratio. **(i)** Growth on same substrate (S^0^) at different points on pH and growth curve (pH 2.5 = day 3/pH 1.5 = day 5), **(ii)** growth to same pH (2.5) on different substrates and points on growth curve (S^0^ = day 3/${\text{S}}_{2}\,{\text{O}}_{3}^{2-}$ = day 5), **(iii)** growth until day 5 on different substrates and to different pH values (S^0^ = pH 1.5/${\text{S}}_{2}\,{\text{O}}_{3}^{2-}$ = pH 2.5). Color scale against each comparison test based on Log2 values; blue = –6 (i.e., numerator expressed less than denominator), white = 0 (i.e., expression equal), red = + 6 (i.e., numerator expressed more than denominator).

To correlate the results of the analysis of sulfur species in the medium with expression of the sulfur metabolism genes using RNA-seq we first needed to generate a more robust genome sequence than the available draft version (Valdes et al., 2011). The published draft genome sequence (GenBank: AFOH01000000) has 164 contigs at low coverage and a total genome size of 3,019,868 bp, which may lead to incomplete transcriptome analyses. For this reason, we re-sequenced the genome of *A. thiooxidans* ATCC 19377 and Table 1 shows the characteristics of the assembly comprising 22 unique contigs and a total of 3,404,101 bp (almost 13% larger than previously published), with the largest contig (2,390,830 bp) spanning 70% of the total sequence. Two contigs have a highly elevated genome coverage, most likely representing circular plasmids. 27 small contigs (size range between 129 and 7,095 bp) carry polymorphic sites and are therefore not counted in the total genome size but included in the GenBank submission. This Whole Genome Shotgun project has been deposited at DDBJ/ENA/GenBank under the accession SZUV00000000. The version described in this paper is version SZUV01000000. A significantly larger fraction of RNA-seq reads (92% for all growth conditions) aligned to our new genome assembly as compared to the previous draft (29–60%) showing that the quality of assembly was greatly improved over the published GenBank record (Table 2). Gene annotation identified all known genes encoding enzymes of sulfur metabolism such as *sdo* (sulfur dioxygenase), the Sox (sulfur oxidation) complex (*soxA*, *soxB*, *soxX*, *soxY*, and *soxZ)*, *sqr* (sulfide quinone reductase), *doxD* (thiosulfate:quinone oxidoreductase), *tetH* (tetrathionate hydrolase), *sor* (sulfur oxygenase reductase), *rhd* (rhodanese), the heterodisulfide reductase (*hdrA*, *hdrB*, and *hdrC)*, *paps* (phosphoadenosine phosphosulfate reductase) and *aps* (ATP sulfurylase) (Figure 3 and Supplementary Tables S1–S4) (Kletzin, 1989, 1992; Griesbeck et al., 2002; Rzhepishevska et al., 2007; Valdes et al., 2008; Quatrini et al., 2009; Valdes et al., 2009; Mangold et al., 2011; You et al., 2011). The genome contains three copies of *sdo*, two operons encoding the Sox complex, two copies of *rhd* and three copies of *hdrA*. The plasmids apparently do not code for genes that are of interest in this context, with the potential exception of a gene for a “divalent metal cation transporter” (MntH), which may have been recruited via a plasmid to manage the high metal ion concentrations in its natural environment.

**TABLE 1:** Assembly and annotation of the *Acidithiobacillus thiooxidans* genome ATCC 19377.

Characteristic	Value
Total genome size	3,404,101 bp
Total number of unique contigs (including two potential circular plasmids)	22
Largest contig	2,390,830 bp
Contigs carrying polymorphisms	27
Average% GC	52.6
Number of tRNA genes	64
Number of rRNA genes	4
Total number of coding sequences	3,505
Number of proteins with known function	2,242
Number of hypothetical proteins	1,263

**TABLE 2:** Comparison of the percentage of concordant pair alignments between RNA-seq data and the new assembly of the *A. thiooxidans* ATCC 19377 genome and the published draft genome with 164 contigs.

Genome	Elemental sulfur pH 2.5	Elemental sulfur pH 1.5	Thiosulfate pH 2.5
44 contigs genome	92.1	92.3	92.4
164 contigs genome	59.5	29.5	56.8

#### Expression Analysis of the Sulfur Metabolism Genes Using RNA-Seq

For transcriptome analysis, we collected total RNA from cultures of *A. thiooxidans* grown on elemental S^0^ and on ${\text{S}}_{2}\,{\text{O}}_{3}^{2-}$ media (three biological replicates) to compare gene expression on two differing oxidation state sulfur substrates. RNA-Seq analysis is a whole genome approach allowing the detection of low and highly expressed genes using the parameter fragments per kilobase of transcript per million mapped reads (FPKM) [Sequence Read Archive (SRA) accession: PRJNA541131]. To assess the quality of mapping of the RNA-Seq sequences on the genome assembly, we compared the percentage of concordant pair alignments using the same raw RNA-Seq data and the two available genomes [our new assembly and the previously published draft genome (Valdes et al., 2011)]. We observed an increase of more than 30% of the total concordant pair alignments of the RNA-Seq data for the newly assembled genome for each individual sample as compared to the draft (Table 2). These data underline the quality of the new genome assembly that was used for all the following analyses. A direct representation of the FPKM values, i.e., relative expression levels for the three growth conditions (exponential and stationary growth phases on S^0^ and stationary growth phase on ${\text{S}}_{2}\,{\text{O}}_{3}^{2-}$) is shown in Figure 3A. FPKM values under 200 are interpreted as low to no expression, as compared to low expression (200–1,000 FPKM), medium expression (1,000–4,000 FPKM), high (4,000–10,000 FPKM), and very highly expressed (more than 10,000 FPKM).

The genes encoding the Sox complex (*soxA*, *B*, *X*, *Y*, *Z*) are generally highly expressed, but interestingly the relative expression of the two *sox* operons changes during growth on elemental sulfur at pH 2.5 (day 3) and pH 1.5 (day 5); *sox-1* strongly decreases and *sox-2* increases to medium levels. In contrast, the *sox-1* operon is very highly expressed during growth on ${\text{S}}_{2}\,{\text{O}}_{3}^{2-}$ and we also observe medium to high expression of the *sox-2* operon showing the importance of the gene products under this condition.

The *sqr* gene is medium to highly expressed in all three conditions at comparable levels suggesting that the gene product sulfide quinone reductase also plays an important role in *A. thiooxidans* S metabolism. Other genes are relatively weakly expressed, and whereas there is some variation of gene expression, it is difficult to assess whether they provide major contributions to sulfur metabolism under these conditions (*aps*, *doxD*, *sor*, and *paps*). We observe low expression of the *rhd* gene and medium to very high expression of *hdrA*, *hdrB*, *hdrC* genes under all conditions. In the case of *sdo*, encoding sulfur dioxygenase required for the entry of elemental sulfur into the cell, the expression of one copy is low under all conditions, whereas two gene copies are below 200 FKPM values (Figure 3A).

#### Further Pairwise Expression Analysis of the Sulfur Metabolism Genes Using RNA-seq

Expression of most of the *A. thiooxidans* sulfur genes (with exceptions of the *sox-2* operon, *hdr*, all but *hdrA-2*, and *paps* genes) was higher on day 3 during exponential growth on S^0^ media, as compared to day 5 when cells were in the stationary phase (Figure 3B-i). It thus appears that *A. thiooxidans* exhibits greater metabolic variability in the genes involved, producing higher oxidation state sulfur species (e.g., polythionates) (Figure 1A), during exponential phase, which shifts during stationary phase to a greater processing of polythionates and decreasing pH values (Figure 2). In addition, *hdrA-1* and *hdrA-3* expression strongly increases at pH 1.5 as compared to pH 2.5 during growth on sulfur, suggesting an increased importance of heterodisulfide reductase in the late growth phase. In contrast, the tetrathionate hydrolase encoding gene (*tetH*) is highly expressed only during stationary growth on thiosulfate (day 5), suggesting that this protein plays a specific role in growth on this SOI compound.

*Acidithiobacillus thiooxidans* gene expression also differed between the two growth media, when an identical pH of 2.5 had been reached. Higher expression levels of the *sox* complex, *tetH*, *hdrA-3*, and *paps* genes were observed for growth on ${\text{S}}_{2}\,{\text{O}}_{3}^{2-}$ (day 5, stationary phase), whilst higher expression levels of the all the *sdo* copies, *sqr*, *rhd-2*, *aps* and all the *hdr* genes except *hdrA-3* were observed during growth on S^0^ (day 3, exponential phase) (Figure 3B-ii). Gene expression levels also differed for day 5 (stationary phase) for *A. thiooxidans* growth in the two sulfur media (Figure 3B-iii): all sulfur genes with the exceptions of *sdo-1, sdo-3, sqr* and all *hdr* genes were more highly expressed when grown on ${\text{S}}_{2}\,{\text{O}}_{3}^{2-}$ compared to growth on S^0^.

#### Genome Wide Analysis of Gene Expression

While the analysis of sulfur genes is vital to the comprehension of autotrophic metabolism, the analysis of the complete transcriptome may lead to the identification of genes that are correlated with this metabolic adaptation. To this effect, we conducted pairwise comparisons of relative gene expression levels (FPKM values) to identify additional up- and down-regulated genes. Analysis of gene expression after growth on elemental sulfur at pH 2.5 compared to pH 1.5 (Supplementary Figure S1a), showed that 20% of the genes (660) are upregulated and 12% (404) are downregulated. The top 50 upregulated genes with the highest degree of differential expression are presented in Supplementary Table S5; several of these genes encode chemotaxis and flagellar components. We also analyzed the top 50 downregulated genes and most encode hypothetical proteins (Supplementary Table S6). Analysis of gene expression after growth on elemental sulfur at pH 2.5 compared to thiosulfate pH 2.5 (Supplementary Figure S1b), shows that 18% (594) are upregulated and 8% (269) are downregulated. The top 50 upregulated genes comprise genes encoding chemotaxis components as well as ATP synthase subunits (Supplementary Table S7). We analyzed the top 50 downregulated genes finding hypothetical proteins as well as transcription factors involved in osmoregulation as well as proteins cytochrome C biogenesis among them (Supplementary Table S8). Finally, analysis of gene expression after growth on thiosulfate at pH 2.5 compared to elemental sulfur at pH 1.5 (Supplementary Figure S1c) shows that 8% of the genes are upregulated (271) and 11% are downregulated (347). The top 50 upregulated genes comprise genes encoding components of cytochrome C biogenesis and of proteins involved in protein folding and outer membrane stability (Supplementary Table S9). Analysis of the top 50 downregulated showed that most encode hypothetical proteins (Supplementary Table S10). Further discussion on these broader metabolic characteristics can be found in Supplementary Text.

### Insights Into Sulfur Pathways Catalyzed by *A. thiooxidans* Grown on S^0^ and ${\text{S}}_{2}\,{\text{O}}_{3}^{2-}$

**FIGURE 4:**
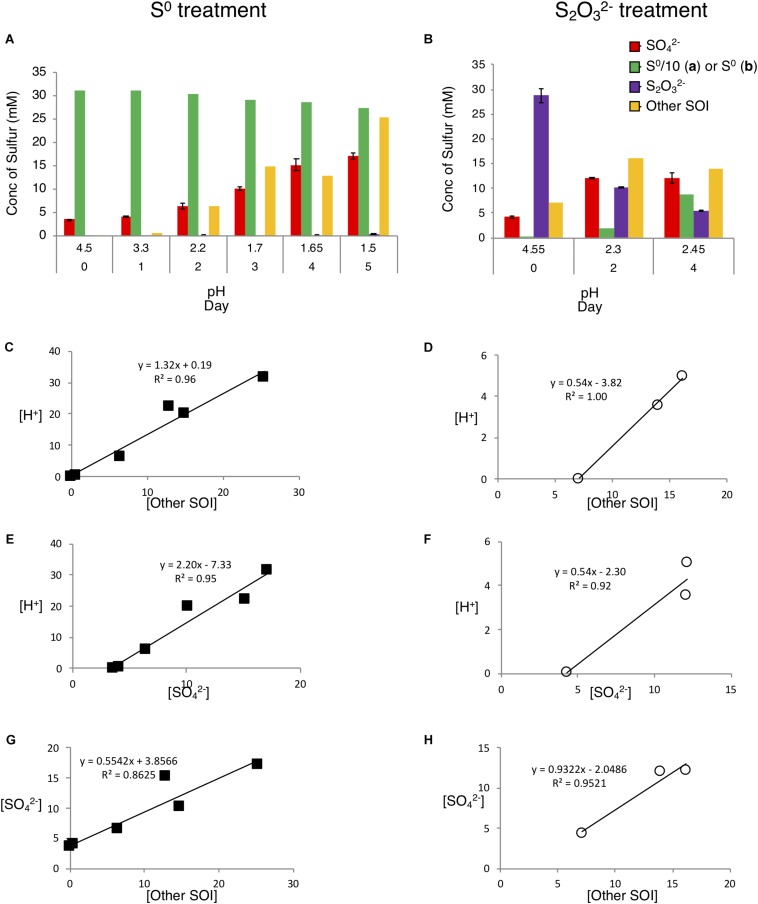
Analysis of sulfur chemistry after growth with S^0^ or ${\text{S}}_{2}\,{\text{O}}_{3}^{2-}$ as energy source. *A. thiooxidans* was cultivated with different substrates for up to 5 days, followed by determination of the production of different sulfur species in the media and comparison of [H^+^] production to S species on S^0^ media (filled squares) and ${\text{S}}_{2}\,{\text{O}}_{3}^{2-}$ media (empty circles). **(A)** Production of sulfur species during growth on S^0^. **(B)** Production of sulfur species during growth on ${\text{S}}_{2}\,{\text{O}}_{3}^{2-}$. **(C,D)** [H^+^] production vs. [Other SOI], **(E,F)** [H^+^] production vs. [${\text{SO}}_{4}^{2-}$], **(G,H)** [${\text{SO}}_{4}^{2-}$ ] production vs. [Other SOI]. The difference in S^0^ concentration scale between figures, where in **(A)** the value shown is in 1/10 actual value. Concentrations of all S species are given in mM in mol of S (e.g., 1 mM of ${\text{SO}}_{4}^{2-}$ = 1 mm S, while 1 mM ${\text{S}}_{2}\,{\text{O}}_{3}^{2-}$ = 2 mM S).

Consistent with the notion that *A. thiooxidans* catalyzes sulfur substrate-dependent metabolic reactions suggested by differential acid production (Figure 2), solution sulfur speciation also differed in the two growth media (Figure 4). *A. thiooxidans* growth on S^0^ resulted in relatively higher concentrations of produced *Other SOI* (i.e., unresolved S species; 25.3 mM versus 6.9 mM on ${\text{S}}_{2}\,{\text{O}}_{3}^{2-}$) and ${\text{SO}}_{4}^{2-}$ (13.7 mM versus 7.8 mM on ${\text{S}}_{2}\,{\text{O}}_{3}^{2-}$; Figures 4A,B), while growth on ${\text{S}}_{2}\,{\text{O}}_{3}^{2-}$ resulted in near equal generation of *Other SOI*, sulfate and S^0^ (Figures 4A,B). Further, S^2–^ and ${\text{SO}}_{3}^{2-}$ were largely non-detectable in solution, with the exception of a very low amount of ${\text{SO}}_{3}^{2-}$ on day 5 in the S^0^ growth experiment (Supplementary Table S11), while both sulfur species were detected at low concentrations (<0.5 mM) throughout growth on ${\text{S}}_{2}\,{\text{O}}_{3}^{2-}$ (Supplementary Table S11).

Sulfur mass balance identified that concentrations of unresolved sulfur species, *Other SOI*, occurred at appreciable levels under both growth conditions (Figures 4A,B). This *Other SOI* pool may variably comprise a number of possible sulfur intermediate oxidation compounds, such as species associated with oxidation pathways, i.e., polythionates, as well as products of disproportionation reactions, i.e., polysulfides. While our results do not identify the specific species sulfur species occurring within this pool, insights provided through analysis of the relationships between changes in (1) [*Other SOI*] and (2) [${\text{SO}}_{4}^{2-}$ ] to [H^+^] (Figures 4C–H), suggest that the unresolved sulfur species differ in their composition between the two growth treatments. The high correlations and statistical significance (*p*-value < 0.05) for Figures 4C–H assist in providing a strong rationale for the basis of stoichiometric reactions occurring in the respective sulfur substrates individual metabolism. The higher slopes observed during growth on S^0^ (Figures 4C,E) alongside the greater overall H^+^ generation (10-fold higher total H^+^ increase) imply greater overall oxidation compared to growth on ${\text{S}}_{2}\,{\text{O}}_{3}^{2-}$ (Figures 4D,F and Supplementary Figure S2a). During growth on S^0^, a decrease in ΔS^0^, and increases in both Δ*Other SOI* and Δ${\text{SO}}_{4}^{2-}$ imply that S^0^ is first converted to higher oxidation state SOI, e.g., polythionate species, and ultimately to ${\text{SO}}_{4}^{2-}$ (Supplementary Figure S2a); consistent with predominantly oxidative (i.e., acid generating) pathways (i.e., Eqs 2–6; Table 3). During growth on ${\text{S}}_{2}\,{\text{O}}_{3}^{2-}$, Δ*Other SOI* and Δ${\text{SO}}_{4}^{2-}$ increase from days 0 to 2, while, Δ*Other SOI* subsequently decreases and Δ${\text{SO}}_{4}^{2-}$ does not change from days 2 to 4 (Supplementary Figure S2b), These results are consistent with oxidative pathways occurring initially (i.e., Eqs 5, 6, 9, and 10; Table 3), followed by disproportionating pathways (e.g., Eq. 11; Table 3; as shown further and in Supplementary Text), reflected in an increase in ΔS^0^. Consistent with a potential shift from oxidative (i.e., greater acid generating) to disproportionating reactions dominating, Δ[H^+^] increased between days 0 and 2, and subsequently decreased from days 2 to 4 (Supplementary Figure S2b).

**TABLE 3:** Mass balance S reactions for the two treatments and potential S abiotic and biotic reactions important for stoichiometric balancing.

Formula	Eq. #	References
$6\,S^{0}\to 2\,S\,O_{4}^{2-}+4\,S^{\text{Other}\cdot \text{SOI}}+5\,H^{+}$	1	This paper, S^0^ treatment days 0–5
${\text{S}}^{0}+O_{2}+H_{2}\,\text{O}\to {\mathrm{SO}}_{3}^{2-}+2\,H^{+}$	2	Based on Suzuki (1999)
${\text{SO}}_{3}^{2-}+0.5\,O_{2}\to {\text{SO}}_{4}^{2-}$	3	Based on Suzuki (1999)
${\text{S}}^{0}+{\mathrm{SO}}_{3}^{2-}↔{\text{S}}_{2}\,{\text{O}}_{3}^{2-}$	4	Based on Johnston and McAmish (1973) and Suzuki (1999)
$2\,S_{2}\,{\text{O}}_{3}^{2-}+0.5\,O_{2}+2\,H^{+}\to {\text{S}}_{4}\,{\text{O}}_{6}^{2-}+H_{2}\,\text{O}$	5	Based on Suzuki (1999)
${\text{S}}_{4}\,{\text{O}}_{6}^{2-}+3.5\,O_{2}+3\,H_{2}\,\text{O}\to 4\,S\,O_{4}^{2-}+6\,H^{+}$	6	Based on Suzuki (1999)
$37\,S_{2}\,{\text{O}}_{3}^{2-}+0.5\,{\mathrm{SO}}_{3}^{2-}\to 7\,S^{0}+31.5\,{\mathrm{SO}}_{4}^{2-}$	7	This paper, ${\text{S}}_{2}\,{\text{O}}_{3}^{2-}$ treatment days 0–2
+ 36S^Other⋅SOI^ + 20H^+^		
$3\,S_{2}\,{\text{O}}_{3}^{2-}\to 2\,S^{0}+2\,S\,O_{4}^{2-}+2\,S^{\text{Other}\cdot \text{SOI}}+H^{+}$	8	This paper, ${\text{S}}_{2}\,{\text{O}}_{3}^{2-}$ treatment days 0–4
${\text{SO}}_{3}^{2-}+H_{2}\,\text{O}\to {\text{SO}}_{4}^{2-}+2\,H^{+}$	9	Based on Suzuki (1999)
${\text{S}}_{4}\,{\text{O}}_{6}^{2-}+H_{2}\,\text{O}\to {\text{S}}_{3}\,{\text{O}}_{3}^{2-}+{\mathrm{SO}}_{4}^{2-}+2\,H^{+}$	10	Based on Pronk et al. (1990) and Suzuki (1999)
$4\,{\text{S}}_{3}\,{\text{O}}_{3}^{2-}\to 8\,S^{0}+4\,S\,O_{3}^{2-}$	11	Based on Steudel et al. (1987) and Pronk et al. (1990)

### Electron Microscopic and Spectroscopic Analyses of Intracellular S^0^ Storage

**FIGURE 5:**
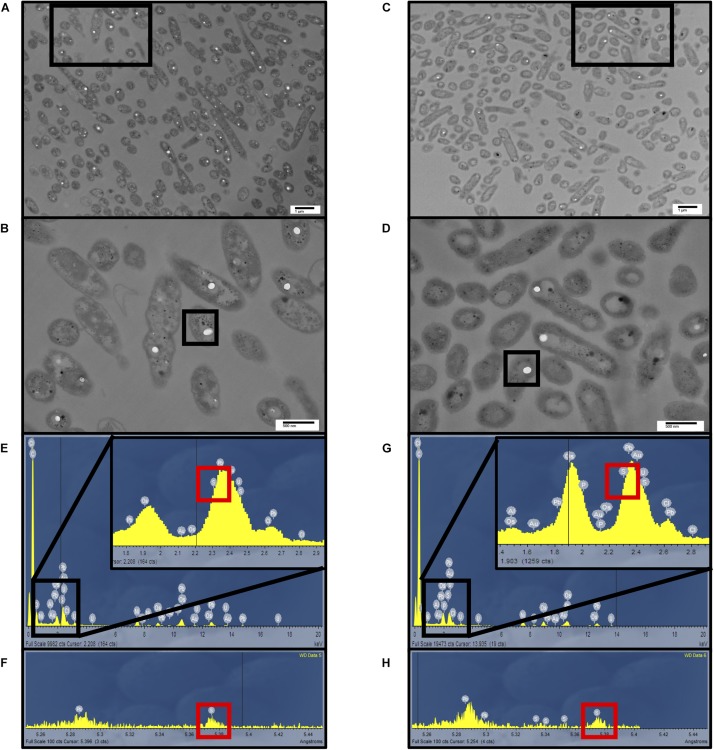
Electron microscopic analysis of sulfur globule formation. Transmission electron microscopy of *A. thiooxidans* cells grown in S^0^ media at pH 1.5 or 2.5 **(A,B)** and ${\text{S}}_{2}\,{\text{O}}_{3}^{2-}$ media at pH 2.5 **(C,D)**, respectively. Scale bars in **(A,C)** indicate 1 μm and 500 nm in **(B,D)**. **(E,G)** Energy-dispersive X-ray spectroscopy (EDS) analysis was conducted on the sulfur globules indicated in **(B,D)**, revealing the presence of different elements shown by their characteristic emission energies. **(F,H)** To better separate the signals, wavelength-dispersive X-ray spectroscopy (WDS) analysis was conducted on the sulfur globules indicated in **(B,D)**, confirming the presence of sulfur.

Transmission electron microscopy in tandem with energy-dispersive X-ray spectroscopy (EDS) and wavelength dispersive spectroscopy (WDS) revealed sulfur globule formation in the cells (Figures 5A–H). The globules did not differ in size (Supplementary Figure S3), but quantification indicated that a higher number (45.6 per 100 bacteria) were observed for *A. thiooxidans* grown on S^0^, while a lower number of internal S^0^ globules (13.5 per 100 bacteria) occurred for *A. thiooxidans* grown on ${\text{S}}_{2}\,{\text{O}}_{3}^{2-}$ (Figures 5A,B vs. C,D), consistent with sulfur speciation and mass balance results (Supplementary Table S12).

### Sulfur Metabolism Models

#### Stoichiometric Sulfur Metabolism Arrays

**FIGURE 6:**
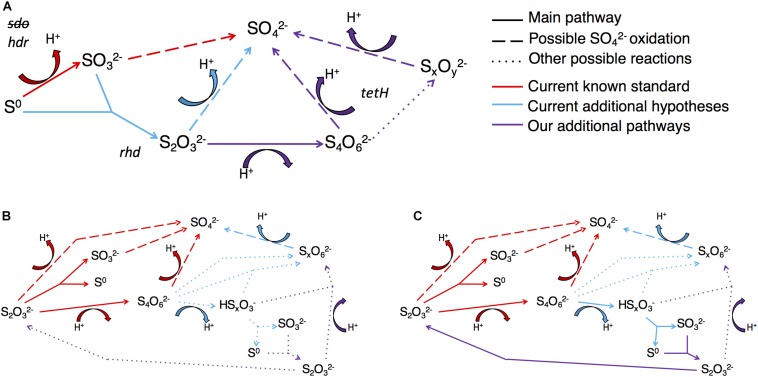
Models of theorized *A. thiooxidans* metabolism for different S reaction pathways, the genes catalyzing them and H^+^ consumption or production reactions based on observed experiments’ mass balance and known potential reactions in literature. **(A)** S^0^ initial reduced S source over total time of experiment (Days 0–5). **(B)** ${\text{S}}_{2}\,{\text{O}}_{3}^{2-}$ initial reduced S source early reactions (days 0–2). **(C)** ${\text{S}}_{2}\,{\text{O}}_{3}^{2-}$ initial reduced S source for total time of experiment (days 0–4). ${\text{SO}}_{3}^{2-}$ can be oxidized both biotically via H_2_O releasing H^+^ and abiotically via O_2_ which is neutral.

We developed *A. thiooxidans* metabolism models by combining observed solution S speciation and [H^+^] changes with FPKM gene expression levels to elucidate the most likely pathways being catalyzed. The generated *A. thiooxidans* S^0^ metabolism model identifies conversion of S^0^ into 1/3 ${\text{SO}}_{4}^{2-}$ and 2/3 S^Other SOI^ [Eq. 1; assumption of initial *Other SOI* generated to be ${\text{S}}_{4}\,{\text{O}}_{6}^{2-}$; the initial metabolism reaction from ${\text{S}}_{2}\,{\text{O}}_{3}^{2-}$ (Eq. 5, Table 3)]. While there are uncertainties as to whether the *Other SOI* pool is solely polythionate species and/or comprises the same polythionates at any given sampling point in either treatment, the highly significant correlations between acid generation and this specific sulfur pool (Figures 4G,H) are consistent with this assumption (Eqs 1, 7, and 8, Figures 6A–C and Table 3, respectively).

(1) $$6\,{\text{S}}^{0}\to 2\,{\text{SO}}_{4}^{2-}+4\,{\text{S}}^{\text{OtherSOI}}+5\,H^{+}$$

Thus our *A. thiooxidans* S^0^ metabolism model identifies the following suite of reactions occur throughout the time course of the experiment (Figure 6A).

(2) $${\text{S}}^{0}+O_{2}+H_{2}\,\text{O}\to {\text{SO}}_{3}^{2-}+2\,H^{+}$$

(3) $${\text{SO}}_{3}^{2-}+0.5\,O_{2}\to {\text{SO}}_{4}^{2-}$$

(4) $$S^{0}+{\mathrm{SO}}_{3}^{2-}↔{\text{S}}_{2}\,{\text{O}}_{3}^{2-}$$

(5) $$2\,S_{2}\,{\text{O}}_{3}^{2-}+0.5\,O_{2}+2\,H^{+}\to {\text{S}}_{4}\,{\text{O}}_{6}^{2-}+H_{2}\,\text{O}$$

The model stoichiometrically balances the observed changes in elemental sulfur concentration. However, the model predicts a greater acid generation than observed. Specifically, the model predicts production of 6H^+^ for every 6S^0^ converted to 2^SO_4^2–^ and 4S (as *Other SOI*); whereas we observe 5H^+^. The same observed lower H^+^ generation relative to expected, also occurs for a model incorporating successive oxidative processing of sulfur by an alternative set of pathways that would exclude ${\text{SO}}_{3}^{2-}$ oxidation to ${\text{SO}}_{4}^{2-}$(Eq. 3), and proceed via oxidation of ${\text{S}}_{2}\,{\text{O}}_{3}^{2-}$ and ${\text{S}}_{4}\,{\text{O}}_{6}^{2-}$ and other polythionates to ${\text{SO}}_{4}^{2-}$(Table 3, Eqs. 5 and 6).

(6) $${\text{S}}_{4}\,{\text{O}}_{6}^{2-}+3.5\,O_{2}+3\,H_{2}\,\text{O}\to 4\,S\,O_{4}^{2-}+6\,H^{+}$$

A stoichiometrically balanced sulfur and H^+^ model of *A. thiooxidans* ${\text{S}}_{2}\,{\text{O}}_{3}^{2-}$ metabolism developed for days 0–2 or for the entire time course of days 0–4 (Table 3; Eqs. 7 and 8, respectively) identifies the most likely occurring reactions would include conversion of ${\text{S}}_{2}\,{\text{O}}_{3}^{2-}$ to ${\text{SO}}_{3}^{2-}$, S^0^ and polythionates (*Other SOI*) and ultimately to ${\text{SO}}_{4}^{2-}$, with the reverse of Eq. 4 followed by Eqs. 3, 5, 6, and 9 as the dominant reactions (Figure 6B) (Table 3).

(7) $$37\,S_{2}\,{\text{O}}_{3}^{2-}+0.5\,{\mathrm{SO}}_{3}^{2-}\to 7\,S^{0}+31.5\,{\mathrm{SO}}_{4}^{2-}+36\,S^{\text{OtherSOI}}+20\,H^{+}$$

(8) $$3\,S_{2}\,{\text{O}}_{3}^{2-}\to 2\,S^{0}+2\,S\,O_{4}^{2-}+2\,S^{\text{Other}\cdot \text{SOI}}+H^{+}$$

(9) $${\text{SO}}_{3}^{2-}+H_{2}\,\text{O}\to {\text{SO}}_{4}^{2-}+2\,H^{+}$$

However, the ${\text{S}}_{2}\,{\text{O}}_{3}^{2-}$ metabolism model of *A. thiooxidans* for days 2–4 indicates disproportionation of ${\text{S}}_{4}\,{\text{O}}_{6}^{2-}$ and ${\text{S}}_{3}\,{\text{O}}_{3}^{2-}$ to S^0^ and ${\text{SO}}_{3}^{2-}$ are occurring (Table 3, Eqs. 10 and 11).

(10) $${\text{S}}_{4}\,{\text{O}}_{6}^{2-}+H_{2}\,\text{O}\to {\text{S}}_{3}\,{\text{O}}_{3}^{2-}+{\mathrm{SO}}_{4}^{2-}+2\,H^{+}$$

(11) $$4\,S_{3}\,{\text{O}}_{3}^{2-}\to 8\,S^{0}+4\,S\,O_{3}^{2-}$$

These disproportionation reactions would recycle sulfur back to ${\text{S}}_{2}\,{\text{O}}_{3}^{2-}$, continuing to consume H^+^ via regenerated reduced SOI species such as ${\text{S}}_{2}\,{\text{O}}_{3}^{2-}$ and S^0^ over the time period of days 2–4. ${\text{S}}_{2}\,{\text{O}}_{3}^{2-}$ model reaction arrays (Figures 6B,C) can also be stoichiometrically balanced via other pathways involving oxidation of polythionates and thiosulfate to sulfate. However, informed by gene expression, results, S metabolism for days 0–2 and days 2–4 is more consistent with the reactions identified above. The most robust model for days 0–4 based on currently theorized/known sulfur reactions follows the series of reactions shown in Figure 6C, identifying the important formation and accumulation of S^0^. Stepwise reactions for Figure 6 are identified in Supplementary Text.

#### Models of *A. thiooxidans* S^0^ and ${\text{S}}_{2}\,{\text{O}}_{3}^{2-}$ Metabolism

**FIGURE 7:**
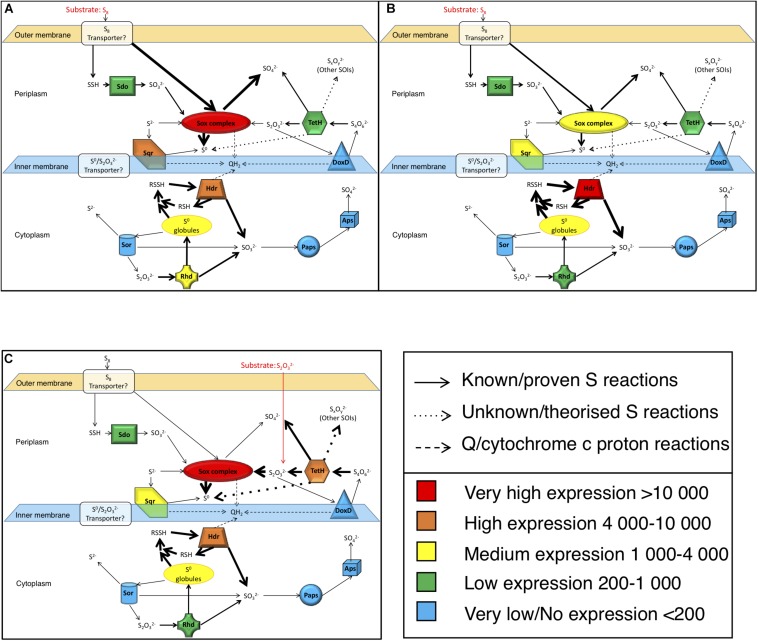
Model of *A. thiooxidans* S metabolism based on the analyses of the expression of key genes encoding S-metabolizing enzymes and of S solution chemistry. Gene expression values are based on the results shown in Figure 3A (FPKM). **(A)** Model for exponential growth phase in S^0^ media (day 3, pH 2.5); **(B)** model for stationary growth phase in S^0^ media (day 5, pH 1.5); **(C)** model for stationary growth phase in ${\text{S}}_{2}\,{\text{O}}_{3}^{2-}$ media (day 5, pH 2.5); models taking results of gene expression analysis and sulfur chemistry into account. Thickness of the arrows reflects importance of the pathways.

By combining the analysis of gene expression, solution sulfur speciation and electron microscopy, our results provide new insights into *A. thiooxidans* sulfur metabolism revealing the importance of intracellular pathways. Based on these data we propose models for the metabolism of *A. thiooxidans* grown on S^0^ (Figures 7A,B) suggesting that the Sox complex plays a major role initiating metabolism after entry of S^0^ into the cell via unknown transporters. There is little published information on the transport of S^0^ into cells to date, however, it has been postulated by other studies to occur via outer membrane proteins (Sugio et al., 1991; Buonfiglio et al., 1999; Rohwerder and Sand, 2003). Sdo is not highly expressed, but it may also contribute to S^0^ metabolism. The intracellular S^0^ is metabolized subsequently through both oxidative and comproportionating pathways. Cytoplasmic Hdr catalyzes S^0^ oxidation generating intracellular ${\text{SO}}_{3}^{2-}$. While it is not certain which gene(s) are involved in intracellular S^0^ comproportionation and buildup of sulfur granules, the high expression of genes responsible for ${\text{SO}}_{3}^{2-}$ production (*hdr*) yet low concentrations in bulk solution, suggest that this pathway generates ${\text{S}}_{2}\,{\text{O}}_{3}^{2-}$. We believe that this pathway is active, because we observe medium-level expression of Rhd known to catalyze ${\text{S}}_{2}\,{\text{O}}_{3}^{2-}$ disproportionation (Figure 7A), possibly acting in a reverse reaction utilizing the high intracellular S^0^ and ${\text{SO}}_{3}^{2-}$ to produce ${\text{S}}_{2}\,{\text{O}}_{3}^{2-}$, which is then oxidized to higher order S species (e.g., tetra- and other polythionates). This possibility is consistent with the observed increased concentration of the *Other SOI* pool and ultimately ${\text{SO}}_{4}^{2-}$ (Figure 4A). These higher oxidation state S species (i.e., ${\text{S}}_{4}\,{\text{O}}_{6}^{2-}$ and/or other higher chain polythionates represented, we believe, in the *Other SOI* fraction based on S speciation, [H^+^] changes and gene expression results presented above) generated through S^0^ comproportionation are oxidized through TetH catalysis resulting in ${\text{SO}}_{4}^{2-}$. The observed increase in Hdr expression from exponential growth (Figure 7A) to stationary growth (Figure 7B) supports the notion that this pathway would catalyze growth through intracellular recycling of sulfur, and implies the synthesis of sulfur storage granules.

The model for growth of *A. thiooxidans* on ${\text{S}}_{2}\,{\text{O}}_{3}^{2-}$ implies that the Sox complex catalyzes ${\text{S}}_{2}\,{\text{O}}_{3}^{2-}$ disproportionation to S^0^ and ${\text{SO}}_{4}^{2-}$ (Figure 7C), while TetH catalyzes oxidation and conversion of ${\text{S}}_{4}\,{\text{O}}_{6}^{2-}$ to other higher chain polythionates [consistent with detection of *Other SOI*, which would include these unresolved S compounds (Figure 4B)]. These higher oxidation S compounds are then disproportionated via the Sox complex and/or TetH catalysis, resulting in intracellular S^0^, and the subsequent intracellular generation of ${\text{SO}}_{3}^{2-}$ indicated to occur by the high level of Hdr expression (Figure 7C). Comproportionation reforming ${\text{S}}_{2}\,{\text{O}}_{3}^{2-}$ from the high intracellular S^0^ and ${\text{SO}}_{3}^{2-}$ catalyzed by Rhd may also be possible, thereby recycling S within the cell. Alternatively, the low levels of expression of DoxD (Figure 3A), suggest that either TetH may be catalyzing a reverse reaction from ${\text{S}}_{2}\,{\text{O}}_{3}^{2-}$ to ${\text{S}}_{4}\,{\text{O}}_{6}^{2-}$ (or to *Other SOI*), or there may be other proteins responsible for ${\text{S}}_{2}\,{\text{O}}_{3}^{2-}$ oxidation to higher chain polythionates.

## Discussion

### Novel Insights Into S-Metabolism: Importance of S^0^, ${\text{S}}_{2}\,{\text{O}}_{3}^{2-}$, ${\text{SO}}_{3}^{2-}$ and Intracellular Reactions

#### Comparisons to Previous Literature

The models of *A. thiooxidans* sulfur metabolism that were developed through integrated analysis of gene expression, sulfur chemistry, sulfur mass balance and electron microscopy reveal new insights into the importance of intracellular reactions involving TetH- and Hdr-catalyzed transformation of S^0^ into ${\text{SO}}_{3}^{2-}$ species, compared to previous models. Bobadilla Fazzini et al. (2013) analyzed the solution concentrations of two sulfur species, S^0^ and ${\text{S}}_{2}\,{\text{O}}_{3}^{2-}$, for *A. thiooxidans* DSM 17318 at stationary phase when grown in S^0^ and ${\text{S}}_{4}\,{\text{O}}_{6}^{2-}$ media at low pH (1.8 and 2.5, respectively). Their chemically based model identified the same comproportionation reaction involving S^0^ and ${\text{SO}}_{3}^{2-}$ to form ${\text{S}}_{2}\,{\text{O}}_{3}^{2-}$ (Eq. 4; Table 3) as identified here for *A. thiooxidans* S^0^ growth. However, they speculated that Sdo was the most important protein for ${\text{SO}}_{3}^{2-}$ production, while our results are more consistent with the Hdr protein catalyzing this reaction. Further, their S^0^ metabolism model does not account for activity of the TetH enzyme, resulting in less S^0^ storage and *Other SOI* (e.g., polythionates) production. Bobadilla Fazzini et al. (2013) also modeled *A. thiooxidans* growth on ${\text{S}}_{4}\,{\text{O}}_{6}^{2-}$ and suggest, based only on their chemical analyses of S^0^ and ${\text{S}}_{2}\,{\text{O}}_{3}^{2-}$ that S^0^ production from polythionates occurs with no involvement of TetH. In contrast, our combined chemical and gene expression results assessing *A. thiooxidans* growth on ${\text{S}}_{2}\,{\text{O}}_{3}^{2-}$, show that TetH is highly expressed (Figure 7C) and associated with the evident production of intracellular S^0^ determined by microscopy and elemental analyses (Figure 5C). This intracellular S^0^ plays a central role in ${\text{S}}_{2}\,{\text{O}}_{3}^{2-}$ metabolism (Figure 6C). The Bobadilla Fazzini et al. (2013) model did not predict any S^0^ storage for *A. thiooxidans* grown on S^0^, or storage in tandem with TetH activity for *A. thiooxidans* grown on ${\text{S}}_{4}\,{\text{O}}_{6}^{2-}$ and did not include the *hdr*, *rhd*, *paps*, and *aps* genes.

Yin et al. (2014) examined *A. thiooxidans* A01 via gene expression proposing a similar sulfur gene model to our *A. thiooxidans* ATCC 19377 model. Due to our improved draft genome of *A. thiooxidans* ATCC 19377, we were able to find and confirm the previously elusive *sor* gene (Valdes et al., 2011) identifying that the same sulfur genes are present in the two strains (Figure 1B). However, differences in the number of gene copies identified for *hdrA*, *rhd*, *paps*, and *aps* exist between the strains, where for *A. thiooxidans* ATCC 19377, we found three copies of *hdrA*, two copies of *rhd* and one copy each for *paps* and *aps* (Figure 3). In contrast, for *A. thiooxidans* A01 one copy of *hdrA*, five copies of *rhd*, three copies of *paps*, and two copies of *aps* were identified (Yin et al., 2014). That study found that most sulfur metabolic genes were more strongly expressed in *A. thiooxidans* A01 when grown on S^0^ compared to ${\text{S}}_{2}\,{\text{O}}_{3}^{2-}$ during exponential growth phase (Yin et al., 2014), showing opposing results to our relative expression levels for *A. thiooxidans* ATCC 19377 stationary phase growth on S^0^ compared to ${\text{S}}_{2}\,{\text{O}}_{3}^{2-}$ (Figure 3B-iii), These results suggest that relative gene expression switches from lower to higher in ${\text{S}}_{2}\,{\text{O}}_{3}^{2-}$, and higher to lower in S^0^, as *A. thiooxidans* goes from exponential to stationary growth phase. However, the Yin et al., 2014 study only examined gene expression during exponential growth phase, and their hypothetic models included pathways identified from previous studies depicting models for other *Acidithiobacillus* species (Yin et al., 2014). Thus, their model was not able to identify the importance of intracellular S^0^, and ${\text{SO}}_{3}^{2-}$ and the *hdr* gene as observed here.

In comparison to other *Acidithiobacillus* species models of S metabolism, our gene model for *A. thiooxidans* shows closest similarity to *A. caldus*. Differing only that in *A. caldus*, Sdo has been determined to be located in the cytoplasm instead of the periplasm (Wu et al., 2017) and it has the addition of SAT responsible for oxidation of sulfite to sulfate (Wang et al., 2019). While the *A. ferrooxidans* gene model shows greater differences to our *A. thiooxidans* model, most notably in its absence of the Sox complex and *sor* gene, and its inclusion of SAT and TSD (Wang et al., 2019; Zhan et al., 2019). For *A. caldus* and *A. ferrooxidans*, the current S^0^ metabolism is proposed to be oxidation to ${\text{SO}}_{3}^{2-}$ via Sdo, followed by oxidation to ${\text{SO}}_{4}^{2-}$ via SAT, with the bacteria acquiring S^0^ from extracellular sources (Mangold et al., 2011; Zhan et al., 2019). This differs to our proposed S^0^ metabolic pathway (Figure 6A), which is elaborated upon further below. The current proposed model of ${\text{S}}_{2}\,{\text{O}}_{3}^{2-}$ oxidation metabolism, shows that both *A. caldus* and *A. ferrooxidans* utilize the S_4_I pathway, however, *A. caldus* also uses the Sox system while *A. ferrooxidans* also uses TSD (Ghosh and Dam, 2009; Wang et al., 2019). Our *A. thiooxidans* S metabolism model follows the same ${\text{S}}_{2}\,{\text{O}}_{3}^{2-}$ oxidation metabolism as *A. caldus* employing both the S_4_I pathway and the Sox system.

#### Integrated Gene Expression and Sulfur Chemistry *A. thiooxidans* Metabolism Models

The models generated here provide new insights into the likely pathways involved in *A. thiooxidans* sulfur metabolism, closing some of the gaps in the current understanding. Specifically, our results identify internal cell S^0^ generation, storage and use, as well as the importance and rapid conversion of ${\text{SO}}_{3}^{2-}$ in these models, both confirming the speculated importance of these two S compounds (Suzuki et al., 1992) and explaining why they have not previously been definitively confirmed by solution chemical characterization alone.

Based on the published studies to date, the first step in microbial S^0^ metabolism is thought to be a relatively linear pathway beginning with oxidation to ${\text{SO}}_{3}^{2-}$, followed by further oxidation to ${\text{SO}}_{4}^{2-}$ (Suzuki et al., 1992; Rohwerder and Sand, 2003). However, here the model developed through combined sulfur chemical and gene expression analyses indicates that ${\text{S}}_{2}\,{\text{O}}_{3}^{2-}$ oxidation/disproportionation reactions are occurring as formation of significant amounts of *Other SOI* (i.e., indicating the presence of polythionates) and small amounts of ${\text{S}}_{2}\,{\text{O}}_{3}^{2-}$ are observed (Figure 4A). Consistent with these pathways, expression specifically of *tetH* and *rhd*, genes known to encode enzymes for ${\text{S}}_{2}\,{\text{O}}_{3}^{2-}$ and polythionate oxidation/disproportionation reactions were being expressed (Figures 7A,B) (Meulenberg et al., 1992; Hallberg et al., 1996; Beller et al., 2006; Rzhepishevska et al., 2007).

Further lending support to these alternative pathways, higher relative expression of the *hdr* genes was observed (Figure 3A), which should result in high levels of ${\text{SO}}_{3}^{2-}$, and thus subsequent high ${\text{SO}}_{4}^{2-}$ values. However, our results here indicate lower values of ${\text{SO}}_{4}^{2-}$ than expected, consistent with recycling of this ${\text{SO}}_{3}^{2-}$ through comproportionating reactions that would generate ${\text{S}}_{2}\,{\text{O}}_{3}^{2-}$ instead. The specific presence of ${\text{S}}_{2}\,{\text{O}}_{3}^{2-}$, despite likely abiotic disproportionation at this low pH < 2, ${\text{SO}}_{3}^{2-}$ (Supplementary Table S11) and activity of *hdr* genes associated with sulfur back reactions, underscore the formation of ${\text{S}}_{2}\,{\text{O}}_{3}^{2-}$ as a critical step in S^0^ metabolism (Figure 6A) generating the precursor to most reactions involving the increased pool of *Other SOI*, e.g., polythionates (Meulenberg et al., 1993; Müller et al., 2004).

The formation of ${\text{S}}_{2}\,{\text{O}}_{3}^{2-}$ from ${\text{SO}}_{3}^{2-}$ comproportionation during *A. thiooxidans* S^0^ metabolism is supported by three lines of evidence. First, the intracellular neutral pH of *A. thiooxidans* (Suzuki et al., 1999) makes the neutrophilic reaction combining S^0^ with ${\text{SO}}_{3}^{2-}$ to form ${\text{S}}_{2}\,{\text{O}}_{3}^{2-}$ favorable (Eq. 4, Table 1). Second, *A. thiooxidans* possesses rhodanese/sulfur transferase (Yin et al., 2014), which may include a rhodanese capable of binding a sulfane group sulfur (e.g., S^0^) to ${\text{SO}}_{3}^{2-}$ to form ${\text{S}}_{2}\,{\text{O}}_{3}^{2-}$ (Hildebrandt and Grieshaber, 2008; Zhang et al., 2013). Third, the metabolic bonding of S^0^ with ${\text{SO}}_{3}^{2-}$ is mediated via the Sox complex, which is highly expressed by *A. thiooxidans* grown on S^0^ (Figures 7A,B). The versatility of the Sox complex would support this pathway (Sauvé et al., 2007; Wang et al., 2019). The gene expression results are consistent with comproportionation, as results here identify that within the Sox complex, SoxYZ (carriers) and SoxAX (binders) are more highly expressed than the oxidizing enzyme (SoxB) in all analyses (Figure 3A).

Metabolic modeling results from growth on ${\text{S}}_{2}\,{\text{O}}_{3}^{2-}$ indicate *A. thiooxidans*${\text{S}}_{2}\,{\text{O}}_{3}^{2-}$ oxidation closely follows the S_4_I pathway proposed in the literature, further suggesting higher oxidation chain polythionate formation (Figures 6B,C and Supplementary Text) (Hallberg et al., 1996; Masau et al., 2001; Müller et al., 2004; Ghosh and Dam, 2009). However, the ability to effectively measure all the possible sulfur species remains an analytical challenge (Houghton et al., 2016), which precludes 100% certainty in our model fitting.

The occurrence of S^0^ within the cells when grown on S^0^, can be attributed to the intake of the sulfur globules from the media via transport enzymes and outer membrane proteins (Rohwerder and Sand, 2003), and/or from SOI cycling through mechanisms such as ${\text{S}}_{2}\,{\text{O}}_{3}^{2-}$ oxidation via the Sox complex when missing SoxCD, a characteristic for sulfur globule formation in bacteria species (Figure 5A) (Steudel et al., 1987; Pronk et al., 1990). However, formation of S^0^ within the cells was also observed when *A. thiooxidans* was grown on ${\text{S}}_{2}\,{\text{O}}_{3}^{2-}$ associated with SOI cycling (Figure 5C), though at lower levels than that observed for *A. thiooxidans* grown on S^0^ (Figures 5A vs. C).

### Relevance of Gene Expression Analysis

#### Relative Expression Levels Between Variable Conditions in S Metabolism

Results assessing relative changes in gene expression identify that the Sox complex, Sqr, Hdr, TetH, and Rhd are important in both S^0^ and ${\text{S}}_{2}\,{\text{O}}_{3}^{2-}$ metabolism by *A. thiooxidans*. While measurements of gene expression does not allow firm conclusions on absolute protein levels or enzyme activities, they do identify specific genes and encoded enzymes likely to be important in a metabolic pathway. Relative levels of expression of these genes however differ between the two sulfur media and between growth stages for S^0^ (Figure 3A). The results illustrate the importance of the Sox complex and of TetH for ${\text{S}}_{2}\,{\text{O}}_{3}^{2-}$ metabolism (Figure 7C). The Sox complex is a very important metabolic enzyme complex during growth on both substrates since it is highly expressed under all conditions (Figure 3A). Gene expression results indicate the *sox*-1 operon is active in cells at less acidic pH values and underscore the geochemical flexibility and viability of the Sox complex as expression of the *sox-*2 operon is used under more acidic conditions and with higher thiosulfate concentrations (Figure 3A). These observations are similar to those reported by others (Zhu et al., 2012; Jones et al., 2014; Yin et al., 2014; Li et al., 2017; Wang et al., 2019), whereby different gene copies of the same enzyme express at differing levels due to environmental parameters. The differential expression of these gene copies (Figure 3A) indicates that gene expression can provide insights into the geochemical conditions associated with sulfur metabolism.

#### Insight Into the Importance of Hdr Toward S Metabolism

Our results identify a key role of Hdr in *A. thiooxidans* ATCC 19377 S^0^ metabolism expanding the understanding of important genes and their roles in *A. thiooxidans* sulfur metabolism. Relatively high *hdr* expression levels were observed under all conditions in comparison to the low levels of *sdo* (Figure 3A). The inclusion of solution chemical data and electron microscopy suggest that Hdr is likely the primary S^0^ oxidizing enzyme rather than Sdo, which was previously identified as important for internal generation of ${\text{SO}}_{3}^{2-}$ and ${\text{S}}_{2}\,{\text{O}}_{3}^{2-}$ during growth on S^0^ (Rohwerder and Sand, 2003; Bobadilla Fazzini et al., 2013; Yin et al., 2014; Koch and Dahl, 2018). Catalysis by Hdr rather than Sdo is energetically more favorable since conversion of S^0^ to ${\text{SO}}_{3}^{2-}$ is a non-quinone/cytochrome metabolic step for Sdo. Thus it would result in a loss of approximately 50% of the available potential energy considering the ΔG of −500 to 550 kJ per mol S in oxidation of S^0^ to ${\text{SO}}_{4}^{2-}$ (Kelly, 1999). In contrast, catalysis of the *hdr* gene also found in *A. caldus* (Mangold et al., 2011), *A. ferrooxidans* (Quatrini et al., 2009) and *A. thiooxidans* A01 (Yin et al., 2014) enables *A. thiooxidans* to metabolize and access this energy. The identification of its role in sulfur metabolism here, may assist taxonomic classification and facilitate better understanding of the potential for sulfur metabolism across all *Acidithiobacilli* (Nuñez et al., 2017; Cao et al., 2018; Koch and Dahl, 2018; Wang et al., 2019) and other sulfur oxidizing microbes.

## Conclusion

Here we are able to provide greater insight into the specific reactions being catalyzed by known sulfur genes and newly highlight the role of Hdr in *A. thiooxidans* sulfur metabolism by integrating gene expression levels with bulk solution S speciation. Our results further confirm the importance specifically of ${\text{S}}_{2}\,{\text{O}}_{3}^{2-}$ and ${\text{SO}}_{3}^{2-}$ in *A. thiooxidans* sulfur metabolism, which have been widely accepted in the literature to be important, though not definitively shown to date prior to this study (Suzuki et al., 1992; Suzuki, 1999; Bobadilla Fazzini et al., 2013). Further, our results generate new insights into the central role of intracellular S^0^ generation, transformation and pathways in both ${\text{S}}_{2}\,{\text{O}}_{3}^{2-}$ and S^0^ metabolism and that ${\text{SO}}_{3}^{2-}$ comproportionation to ${\text{S}}_{2}\,{\text{O}}_{3}^{2-}$ is a critical step in S^0^ metabolism. Collectively these results highlight how the integration of molecular biology and chemistry approaches can better inform our understanding of biogeochemical cycling of sulfur by microbes.

## Data Availability Statement

The datasets generated for this study can be found in NCBI GenBank and NCBI Short Read Archive (SRA) repository, submitted and accession number for DNA is SZUV00000000, the accession number for RNA is PRJNA541131.

## Author Contributions

DC and RF did all experimentation, analyses, and wrote manuscript. AF did TEM, EDS, and WDS work. SA provided analyses on ΣS via ICP-AES. AN, BL, CB, and LW provided funding and laboratory expertise. CB and LW also provided manuscript edits and were main supervisors to this work.

## Conflict of Interest

The authors declare that the research was conducted in the absence of any commercial or financial relationships that could be construed as a potential conflict of interest.

## Footnotes

1 https://usegalaxy.org/

2 https://imagej.nih.gov/ij/
