# Supplementary material for: New Insights Into Acidithiobacillus thiooxidans Sulfur Metabolism Through Coupled Gene Expression, Solution Chemistry, Microscopy, and Spectroscopy Analyses
Source: Front Microbiol. 2020 Mar 13;11:411. doi: 10.3389/fmicb.2020.00411 (PMC7082400; doi:10.3389/fmicb.2020.00411)
Supplement: Supplementary file 1 [file Data_Sheet_1.PDF]

Supplementary Table S1: More statistical detailed table of Figure 3a, showing analysis of gene expression after growth with S^0 or $S_2O_3^{2-}$ as energy source, with gene expression based on FPKM values and standard deviation on the biological triplicate experiments.

Gene	Gene number	S^0 pH=2.5	S^0 pH=1.5	$S_2O_3^{2-}$ pH=2.5
sdo-1	PROKKA-ALT_00302	82 ± 9	65 ± 17	57 ± 13
sdo-2	PROKKA-ALT_03084	336 ± 34	265 ± 71	297 ± 1
sdo-3	PROKKA-ALT_00833	122 ± 42	40 ± 11	34 ± 1
soxA-1	PROKKA-ALT_01532	8991 ± 650	740 ± 91	27741 ± 776
soxB-1	PROKKA-ALT_01530	6620 ± 519	506 ± 96	18732 ± 1793
soxX-1	PROKKA-ALT_01535	13321 ± 1082	1431 ± 57	48108 ± 233
soxY-1	PROKKA-ALT_01534	18029 ± 1609	1887 ± 81	58197 ± 1804
soxZ-1	PROKKA-ALT_01533	20503 ± 1519	2138 ± 139	63386 ± 2778
soxA-2	PROKKA-ALT_02661	137 ± 8	970 ± 98	1445 ± 125
soxB-2	PROKKA-ALT_02657	453 ± 5	1358 ± 16	3489 ± 181
soxX-2	PROKKA-ALT_02660	126 ± 4	776 ± 106	1307 ± 102
soxY-2	PROKKA-ALT_02655	597 ± 28	1552 ± 67	4122 ± 113
soxZ-2	PROKKA-ALT_02656	723 ± 21	1764 ± 19	5215 ± 222
sqr	PROKKA-ALT_02961	4623 ± 1085	3353 ± 609	3124 ± 57
doxD	PROKKA-ALT_02168	42 ± 0	39 ± 14	54 ± 13
tetH	PROKKA-ALT_02172	251 ± 80	214 ± 98	5968 ± 1143
sor	PROKKA-ALT_00671	21 ± 2	17 ± 0	26 ± 1
rhd-1	PROKKA-ALT_01651	756 ± 189	839 ± 337	916 ± 42
rhd-2	PROKKA-ALT_01545	1345 ± 182	661 ± 134	702 ± 77
hdrA-1	PROKKA-ALT_01550	8668 ± 607	11964 ± 2479	8236 ± 666
hdrA-2	PROKKA-ALT_00063	50 ± 6	27 ± 11	20 ± 3
hdrA-3	PROKKA-ALT_01357	4628 ± 670	19041 ± 3595	15625 ± 2227
hdrB	PROKKA-ALT_01507	1176 ± 329	1354 ± 124	831 ± 68
hdrC	PROKKA-ALT_01552	4903 ± 488	6565 ± 1594	4219 ± 190
paps	PROKKA-ALT_03389	20 ± 1	80 ± 5	148 ± 29
aps	PROKKA-ALT_02667	178 ± 11	68 ± 25	82 ± 14

Supplementary Table S2: More statistical detailed table of Figure 3b-i, showing analysis of gene expression after growth with S^0 or $S_2O_3^{2-}$ as energy source, with comparative gene expression for FPKM values based on Log2 ratio and p-value based on independent t-test between the respective triplicate samples of growth on same substrate (S^0) at different points on pH and growth curve (pH 2.5 = day 3 / pH 1.5 = day 5).

Gene	Gene number	Log2	Err. standard log2	p-value
sdo-1	PROKKA-ALT_00302	0.33	0.12	2.09E-01
sdo-2	PROKKA-ALT_03084	0.34	0.13	3.34E-01
sdo-3	PROKKA-ALT_00833	1.62	1.01	2.96E-02
soxA-1	PROKKA-ALT_01532	3.60	0.71	3.15E-03
soxB-1	PROKKA-ALT_01530	3.71	0.99	3.70E-03
soxX-1	PROKKA-ALT_01535	3.22	0.39	4.12E-03
soxY-1	PROKKA-ALT_01534	3.26	0.43	4.94E-03
soxZ-1	PROKKA-ALT_01533	3.26	0.45	3.43E-03
soxA-2	PROKKA-ALT_02661	-2.83	-0.86	1.43E-04
soxB-2	PROKKA-ALT_02657	-1.58	-0.04	1.70E-04
soxX-2	PROKKA-ALT_02660	-2.62	-0.71	4.75E-04
soxY-2	PROKKA-ALT_02655	-1.38	-0.12	2.87E-03
soxZ-2	PROKKA-ALT_02656	-1.29	-0.05	3.75E-04
sqr	PROKKA-ALT_02961	0.46	0.19	1.52E-01
doxD	PROKKA-ALT_02168	0.10	0.03	7.54E-01
tetH	PROKKA-ALT_02172	0.23	0.18	6.35E-01
sor	PROKKA-ALT_00671	0.34	0.04	6.26E-03
rhd-1	PROKKA-ALT_01651	-0.15	-0.10	7.30E-01
rhd-2	PROKKA-ALT_01545	1.03	0.35	6.29E-03
hdrA-1	PROKKA-ALT_01550	-0.46	-0.13	8.89E-02
hdrA-2	PROKKA-ALT_00063	0.91	0.49	3.22E-02
hdrA-3	PROKKA-ALT_01357	-2.04	-0.68	2.41E-03
hdrB	PROKKA-ALT_01507	-0.20	-0.08	4.29E-01
hdrC	PROKKA-ALT_01552	-0.42	-0.13	1.59E-01
paps	PROKKA-ALT_03389	-1.97	-0.18	3.29E-03
aps	PROKKA-ALT_02667	1.39	0.61	2.31E-03

Supplementary Table S3: More statistical detailed table of Figure 3b-ii, showing analysis of gene expression after growth with S^0 or $S_2O_3^{2-}$ as energy source, with comparative gene expression for FPKM values based on Log2 ratio and p-value based on independent t-test between the respective triplicate samples of growth to same pH (2.5) on different substrates and points on growth curve (S^0 = day 3 / $S_2O_3^{2-}$ = day 5).

Gene	Gene number	Log2	Err. standard log2	p-value
sdo-1	PROKKA-ALT_00302	0.54	0.18	4.58E-02
sdo-2	PROKKA-ALT_03084	0.18	0.02	2.49E-01
sdo-3	PROKKA-ALT_00833	1.83	0.69	2.15E-02
soxA-1	PROKKA-ALT_01532	-1.63	-0.16	1.45E-03
soxB-1	PROKKA-ALT_01530	-1.50	-0.26	1.04E-02
soxX-1	PROKKA-ALT_01535	-1.85	-0.16	5.05E-04
soxY-1	PROKKA-ALT_01534	-1.69	-0.20	1.81E-03
soxZ-1	PROKKA-ALT_01533	-1.63	-0.19	2.72E-03
soxA-2	PROKKA-ALT_02661	-3.40	-0.99	6.10E-05
soxB-2	PROKKA-ALT_02657	-2.95	-0.19	2.16E-04
soxX-2	PROKKA-ALT_02660	-3.37	-0.72	3.90E-05
soxY-2	PROKKA-ALT_02655	-2.79	-0.21	5.47E-04
soxZ-2	PROKKA-ALT_02656	-2.85	-0.21	1.23E-03
sqr	PROKKA-ALT_02961	0.57	0.14	7.51E-02
doxD	PROKKA-ALT_02168	-0.36	-0.09	1.84E-01
tetH	PROKKA-ALT_02172	-4.57	-2.34	9.87E-04
sor	PROKKA-ALT_00671	-0.28	-0.04	1.28E-01
rhd-1	PROKKA-ALT_01651	-0.28	-0.08	2.27E-01
rhd-2	PROKKA-ALT_01545	0.94	0.23	4.83E-03
hdrA-1	PROKKA-ALT_01550	0.07	0.01	4.53E-01
hdrA-2	PROKKA-ALT_00063	1.32	0.38	1.70E-03
hdrA-3	PROKKA-ALT_01357	-1.76	-0.50	1.21E-03
hdrB	PROKKA-ALT_01507	0.50	0.18	1.51E-01
hdrC	PROKKA-ALT_01552	0.22	0.03	8.65E-02
paps	PROKKA-ALT_03389	-2.86	-0.66	2.54E-02
aps	PROKKA-ALT_02667	1.12	0.26	7.80E-04

Supplementary Table S4: More statistical detailed table of Figure 3b-iii, showing analysis of gene expression after growth with S^0 or $S_2O_3^{2-}$ as energy source, with comparative gene expression for FPKM values based on Log2 ratio and p-value based on independent t-test between the respective triplicate samples of growth until day 5 on different substrates and to different pH values (S^0 = pH 1.5 / $S_2O_3^{2-}$ = pH 2.5).

Gene	Gene number	Log2	Err. standard log2	p-value
sdo-1	PROKKA-ALT_00302	0.20	0.10	5.29E-01
sdo-2	PROKKA-ALT_03084	-0.16	-0.04	5.95E-01
sdo-3	PROKKA-ALT_00833	0.22	0.07	4.47E-01
soxA-1	PROKKA-ALT_01532	-5.23	-0.79	4.19E-04
soxB-1	PROKKA-ALT_01530	-5.21	-1.48	4.82E-03
soxX-1	PROKKA-ALT_01535	-5.07	-0.23	1.30E-05
soxY-1	PROKKA-ALT_01534	-4.95	-0.37	5.14E-04
soxZ-1	PROKKA-ALT_01533	-4.89	-0.53	1.03E-03
soxA-2	PROKKA-ALT_02661	-0.58	-0.11	6.58E-03
soxB-2	PROKKA-ALT_02657	-1.36	-0.09	3.63E-03
soxX-2	PROKKA-ALT_02660	-0.75	-0.16	3.37E-03
soxY-2	PROKKA-ALT_02655	-1.41	-0.10	1.31E-03
soxZ-2	PROKKA-ALT_02656	-1.56	-0.08	2.08E-03
sqr	PROKKA-ALT_02961	0.10	0.02	5.51E-01
doxD	PROKKA-ALT_02168	-0.45	-0.27	2.55E-01
tetH	PROKKA-ALT_02172	-4.80	-3.12	9.67E-04
sor	PROKKA-ALT_00671	-0.62	-0.02	7.13E-04
rhd-1	PROKKA-ALT_01651	-0.13	-0.06	7.15E-01
rhd-2	PROKKA-ALT_01545	-0.09	-0.03	6.68E-01
hdrA-1	PROKKA-ALT_01550	0.54	0.16	6.56E-02
hdrA-2	PROKKA-ALT_00063	0.41	0.24	3.78E-01
hdrA-3	PROKKA-ALT_01357	0.29	0.09	2.34E-01
hdrB	PROKKA-ALT_01507	0.70	0.12	3.03E-03
hdrC	PROKKA-ALT_01552	0.64	0.18	6.47E-02
paps	PROKKA-ALT_03389	-0.89	-0.23	8.39E-02
aps	PROKKA-ALT_02667	-0.27	-0.15	4.47E-01

Supplementary Table S5: Top 50 upregulated genes after growth on elemental sulfur at pH 2.5 as compared to pH 1.5.

p-value	FPKM	LOG2	Genes
2.71E-01	42.98	8.78	16S ribosomal RNA
1.11E-01	4926.88	6.15	putative globin-like protein aq_211
2.15E-01	48.31	5.71	16S ribosomal RNA
7.19E-02	735.70	5.00	Xylene monooxygenase electron transfer component
7.70E-05	743.07	4.68	hypothetical protein
1.13E-02	690.12	4.45	Chemotaxis protein CheY
2.44E-02	339.73	4.38	STAS domain protein
4.08E-02	101.34	4.12	hypothetical protein
8.49E-03	792.53	4.01	Methyl-accepting chemotaxis protein 3
4.47E-02	4950.47	3.99	hypothetical protein
4.05E-01	599.51	3.90	tRNA-Lys(ttt)
2.26E-02	374.81	3.88	Chemotaxis protein CheA
4.07E-01	6.51	3.81	hypothetical protein
2.09E-02	199.92	3.72	Chemotaxis response regulator protein-glutamate methylesterase 2
3.79E-03	1123.46	3.72	Flagellar hook protein FlgE
2.48E-02	409.85	3.56	Biofilm dispersion protein BdlA
4.14E-01	6540.13	3.54	tRNA-Trp(cca)
4.48E-03	1169.28	3.54	Basal-body rod modification protein FlgD
4.51E-02	452.51	3.52	DNA-3-methyladenine glycosylase
2.61E-02	576.58	3.52	Methyl-accepting chemotaxis serine transducer
5.39E-03	1059.93	3.46	hypothetical protein
1.40E-05	189.60	3.43	Flagellar biosynthetic protein FliR
1.42E-02	1023.61	3.42	hypothetical protein
1.82E-03	724.00	3.41	Flagellar biosynthetic protein FliP
6.17E-03	561.07	3.38	Chemotaxis protein CheA
2.46E-02	549.99	3.36	putative chemoreceptor glutamine deamidase CheD
1.56E-02	1065.97	3.35	Flagellar basal-body rod protein FlgC
1.17E-02	439.38	3.33	hypothetical protein
4.23E-02	874.01	3.28	Cytochrome bd-I ubiquinol oxidase subunit 2
1.00E-05	160.77	3.27	Flagellar biosynthetic protein FlhB
8.33E-03	554.28	3.26	hypothetical protein
4.69E-01	287.73	3.21	tRNA-Pro(ggg)
8.23E-03	833.92	3.21	Metallo-beta-lactamase superfamily protein
6.19E-03	1142.73	3.19	Chemotaxis protein CheY
1.66E-02	1435.00	3.18	Flagellar basal body rod protein FlgB
1.03E-02	772.58	3.18	FlgN protein
1.56E-02	839.46	3.17	Flagellar basal-body rod protein FlgF
7.00E-02	1220.94	3.16	Cytochrome bd-II ubiquinol oxidase subunit 1
1.25E-02	238.24	3.13	putative signaling protein
4.74E-01	455.44	3.07	tRNA-Gln(ttg)
9.79E-04	286.66	3.07	Flagellar brake protein YcgR
2.16E-02	1347.68	3.06	hypothetical protein
1.01E-02	1017.38	3.05	hypothetical protein
1.69E-03	1391.39	3.05	flagellar protein FliS
4.67E-02	312.68	3.04	Chemotaxis protein methyltransferase 3
7.09E-02	695.87	3.02	Cytochrome c biogenesis protein CcsB
4.37E-03	2725.95	2.99	hypothetical protein
6.46E-03	440.43	2.99	Flagellar hook-length control protein
1.90E-02	413.96	2.98	Chemotaxis protein CheV
1.26E-01	12.18	2.98	Glycosyl transferase family 2

Supplementary Table S6: Top 50 downregulated genes after growth on elemental sulfur at pH 2.5 as compared to pH 1.5.

p-value	FPKM	LOG2	Genes
3.61E-01	26.96	-6.55	hypothetical protein
3.66E-01	129.99	-6.26	hypothetical protein
3.68E-01	90.04	-6.07	hypothetical protein
3.67E-01	70.83	-6.05	hypothetical protein
3.67E-01	75.00	-6.04	hypothetical protein
3.67E-01	42.30	-5.98	hypothetical protein
3.68E-01	72.61	-5.96	hypothetical protein
3.67E-01	63.09	-5.95	hypothetical protein
3.68E-01	46.96	-5.94	hypothetical protein
3.65E-01	42.37	-5.92	hypothetical protein
3.68E-01	67.87	-5.90	hypothetical protein
3.65E-01	55.69	-5.89	hypothetical protein
3.68E-01	112.53	-5.86	hypothetical protein
3.66E-01	49.85	-5.86	hypothetical protein
3.64E-01	41.91	-5.86	hypothetical protein
3.66E-01	60.91	-5.82	hypothetical protein
3.67E-01	98.17	-5.82	hypothetical protein
3.65E-01	204.11	-5.82	hypothetical protein
3.69E-01	60.88	-5.81	hypothetical protein
3.70E-01	254.82	-5.80	hypothetical protein
3.67E-01	86.34	-5.80	hypothetical protein
3.68E-01	37.68	-5.79	hypothetical protein
3.67E-01	52.53	-5.78	Nucleoid occlusion protein
3.66E-01	71.47	-5.77	hypothetical protein
3.68E-01	26.08	-5.76	hypothetical protein
3.69E-01	38.62	-5.74	hypothetical protein
3.67E-01	57.77	-5.74	hypothetical protein
3.69E-01	85.93	-5.71	Single-stranded DNA-binding protein
3.68E-01	96.85	-5.70	hypothetical protein
3.67E-01	40.19	-5.67	hypothetical protein
3.66E-01	47.84	-5.65	hypothetical protein
3.66E-01	55.51	-5.65	hypothetical protein
3.68E-01	37.68	-5.62	hypothetical protein
3.67E-01	40.47	-5.61	hypothetical protein
3.66E-01	79.07	-5.59	hypothetical protein
3.66E-01	56.27	-5.59	hypothetical protein
3.68E-01	127.91	-5.58	hypothetical protein
3.63E-01	11.50	-5.55	hypothetical protein
3.69E-01	76.44	-5.52	hypothetical protein
3.64E-01	59.73	-5.48	ThiF family protein
3.69E-01	65.72	-5.43	Calcineurin-like phosphoesterase superfamily domain protein
3.69E-01	66.37	-5.41	hypothetical protein
3.68E-01	40.16	-5.40	hypothetical protein
3.62E-01	17.67	-5.37	hypothetical protein
3.67E-01	85.81	-5.36	hypothetical protein
3.65E-01	247.11	-5.35	hypothetical protein
3.71E-01	32.22	-5.21	hypothetical protein
3.68E-01	110.20	-5.20	hypothetical protein
3.69E-01	58.58	-5.15	DNA polymerase III subunit beta
3.64E-01	42.33	-5.14	hypothetical protein

Supplementary Table S7: Top 50 upregulated genes after growth on elemental sulfur at pH 2.5 as compared to thiosulfate pH 2.5.

p-value	FPKM	LOG2	Genes
1.61E-02	365.21	4.04	hypothetical protein
1.91E-03	1656.05	4.02	hypothetical protein
6.80E-03	1593.95	3.94	NADH-quinone oxidoreductase subunit L
3.61E-01	151.44	3.71	putative multidrug resistance protein EmrY
1.18E-03	304.67	3.59	hypothetical protein
3.72E-03	223.33	3.42	Nodulation protein NolG
1.19E-04	799.66	3.41	hypothetical protein
6.22E-03	1336.96	3.36	CobQ/CobB/MinD/ParA nucleotide binding domain protein
4.20E-01	2452.55	3.35	tRNA-Thr(ggt)
1.08E-02	3169.29	3.32	hypothetical protein
5.43E-03	296.13	3.27	hypothetical protein
8.36E-02	1019.12	3.19	hypothetical protein
1.88E-02	929.70	3.16	hypothetical protein
8.45E-03	459.09	3.10	hypothetical protein
4.87E-03	232.78	3.08	hypothetical protein
2.90E-05	212.04	3.05	hypothetical protein
6.80E-02	1058.54	3.02	hypothetical protein
1.67E-02	690.12	3.01	Chemotaxis protein CheY
2.88E-02	374.81	3.00	Chemotaxis protein CheA
3.45E-02	339.73	2.98	STAS domain protein
3.16E-01	0.31	2.94	Rhodopirellula transposase
2.33E-01	236.32	2.94	Cytochrome bd-II ubiquinol oxidase subunit 2
1.47E-02	2695.43	2.94	50S ribosomal protein L7/L12
1.41E-02	276.06	2.89	hypothetical protein
5.46E-04	743.07	2.74	hypothetical protein
3.44E-02	576.58	2.74	Methyl-accepting chemotaxis serine transducer
3.45E-01	72.11	2.72	Fusaric acid resistance protein FusA
4.57E-03	377.74	2.71	hypothetical protein
4.65E-03	127.69	2.67	hypothetical protein
2.20E-01	270.40	2.64	Blue copper oxidase CueO
3.40E-03	796.31	2.63	ATP synthase gamma chain
5.35E-03	754.11	2.56	ATP synthase epsilon chain
7.27E-03	3106.66	2.55	50S ribosomal protein L10
1.60E-02	792.53	2.52	Methyl-accepting chemotaxis protein 3
2.19E-01	132.59	2.51	2-isopropylmalate synthase
4.62E-01	303.63	2.51	tRNA-Val(cac)
2.22E-01	216.52	2.50	Cytochrome bd ubiquinol oxidase subunit 1
5.96E-03	2248.89	2.50	TraT complement resistance protein
2.57E-03	1193.21	2.48	ATP synthase subunit alpha
1.39E-04	184.35	2.48	Capsule polysaccharide export outer membrane protein CtrA
3.65E-03	1318.97	2.44	ATP synthase subunit beta
7.61E-02	12.18	2.37	Glycosyl transferase family 2
7.36E-02	36.98	2.34	RNA polymerase sigma factor RpoS
2.39E-03	801.26	2.33	ATP synthase subunit delta
5.19E-02	124.94	2.33	ATP-binding protein BexA
6.48E-03	660.05	2.23	DNA-directed RNA polymerase subunit beta
1.37E-01	439.74	2.22	Cytochrome bd ubiquinol oxidase subunit 1
3.33E-02	96.75	2.21	Capsule polysaccharide export inner-membrane protein CtrC
1.27E-02	693.33	2.20	DNA-directed RNA polymerase subunit beta'
3.47E-03	1481.47	2.18	ATP synthase subunit b

Supplementary Table S8: Top 50 downregulated genes after growth on elemental sulfur at pH 2.5 as compared to thiosulfate pH 2.5.

p-value	FPKM	LOG2	Genes
1.31E-01	0.11	-9.40	hypothetical protein
1.28E-01	0.17	-8.84	hypothetical protein
8.30E-05	13.89	-5.74	putative protein
1.57E-04	23.79	-5.00	Cation efflux system protein CusC
1.06E-02	24.27	-4.92	hypothetical protein
1.05E-04	30.52	-4.72	putative transporter
1.40E-01	3.91	-3.88	23S ribosomal RNA
4.37E-02	217.63	-3.76	hypothetical protein
1.46E-02	13.92	-3.76	Osmolarity sensor protein EnvZ
1.65E-03	150.95	-3.70	hypothetical protein
5.40E-05	4.67	-3.62	hypothetical protein
2.14E-04	407.47	-3.58	hypothetical protein
3.75E-03	33.17	-3.48	hypothetical protein
6.00E-02	117.39	-3.46	Transcriptional regulatory protein OmpR
3.35E-04	5.98	-3.36	hypothetical protein
6.80E-05	0.19	-3.28	Protein TolB
3.70E-05	203.27	-3.22	Transcriptional regulatory protein OmpR
7.37E-03	179.64	-3.18	hypothetical protein
2.37E-04	169.94	-3.17	Cytochrome c biogenesis protein CcsA
3.51E-04	203.15	-3.17	hypothetical protein
1.59E-02	51.46	-3.15	hypothetical protein
2.35E-03	75.13	-3.12	Cytochrome c biogenesis protein CcsB
4.40E-05	7.24	-3.10	ISXO2-like transposase domain protein
2.45E-03	14.92	-2.91	hypothetical protein
1.78E-03	16.08	-2.74	hypothetical protein
9.76E-04	7.01	-2.65	Outer membrane lipoprotein Slp family protein
1.72E-02	70.10	-2.64	hypothetical protein
1.36E-03	0.06	-2.60	Penicillin-binding protein 1A
2.70E-03	486.91	-2.56	hypothetical protein
3.95E-02	665.72	-2.51	hypothetical protein
2.17E-02	21.72	-2.50	Multidrug resistance protein MdtA
9.33E-03	2669.52	-2.46	hypothetical protein
1.12E-04	7.91	-2.46	hypothetical protein
2.47E-03	1446.84	-2.45	Cytochrome c biogenesis protein CcsA
4.79E-02	26.96	-2.44	hypothetical protein
4.59E-03	30.35	-2.43	hypothetical protein
7.70E-04	10.36	-2.43	putative parvulin-type peptidyl-prolyl cis-trans isomerase
3.11E-04	2.42	-2.42	Outer membrane protein assembly factor BamD
4.62E-03	894.49	-2.42	hypothetical protein
3.37E-02	1.48	-2.40	Multidrug resistance protein MdtA
1.31E-02	49.10	-2.40	Thymidylate kinase
6.36E-04	9.44	-2.38	hypothetical protein
2.91E-03	4035.40	-2.38	DsrE/DsrF-like family protein
3.67E-03	1222.25	-2.38	Thiol:disulfide interchange protein DsbG
5.23E-02	0.15	-2.36	Multidrug resistance protein MdtB
5.38E-03	9.80	-2.32	hypothetical protein
5.11E-03	11.93	-2.32	Protein CyaE
1.56E-03	2.56	-2.32	hypothetical protein
3.90E-02	0.76	-2.31	hypothetical protein
2.11E-03	90.42	-2.31	hypothetical protein

Supplementary Table S9: Top 50 upregulated genes after growth on thiosulfate at pH 2.5 as compared to elemental sulfur pH 1.5.

p-value	FPKM	LOG2	Genes
2.93E-01	62.68	9.32	16S ribosomal RNA
1.32E-01	76.35	7.25	hypothetical protein
1.73E-01	6597.13	6.57	putative globin-like protein aq_211
8.00E-05	740.67	6.35	putative protein
2.97E-01	65.53	6.15	16S ribosomal RNA
1.36E-03	17859.50	5.84	hypothetical protein
1.35E-01	1136.18	5.62	Xylene monooxygenase electron transfer component
1.02E-02	734.44	5.48	hypothetical protein
1.50E-04	758.90	5.45	Cation efflux system protein CusC
1.00E-04	802.89	5.19	putative transporter
3.85E-03	14719.90	4.65	hypothetical protein
9.26E-04	20973.40	4.52	DsrE/DsrF-like family protein
1.37E-02	188.27	4.17	Osmolarity sensor protein EnvZ
4.14E-02	2952.08	4.15	hypothetical protein
1.45E-01	680.51	4.11	DNA-3-methyladenine glycosylase
4.52E-03	1178.45	3.91	ABC transporter, phosphonate, periplasmic substrate-binding protein
3.44E-03	369.92	3.90	hypothetical protein
5.69E-02	1291.91	3.78	Transcriptional regulatory protein OmpR
1.49E-03	6352.36	3.64	Thiol:disulfide interchange protein DsbG
8.47E-04	7884.33	3.53	Cytochrome c biogenesis protein CcsA
8.97E-04	2678.03	3.27	hypothetical protein
4.50E-05	1895.24	3.26	Transcriptional regulatory protein OmpR
1.29E-03	819.75	3.26	Cytochrome c biogenesis protein CcsB
2.13E-04	338.77	3.19	Osmolarity sensor protein EnvZ
1.47E-03	115.31	3.13	Lipoprotein-releasing system ATP-binding protein LolD
4.10E-04	4880.44	3.10	hypothetical protein
4.90E-05	960.76	3.08	Cytochrome c biogenesis protein CcsA
1.79E-01	478.14	3.01	putative protein YeaO
8.70E-04	4131.53	2.96	hypothetical protein
2.75E-03	766.06	2.92	Thiol:disulfide interchange protein DsbD
4.12E-03	49.04	2.86	hypothetical protein
3.31E-01	3.34	2.85	hypothetical protein
4.86E-01	280.35	2.80	tRNA-Lys(ttt)
4.99E-03	171.98	2.71	Transcriptional regulatory protein ZraR
2.83E-04	225.84	2.69	hypothetical protein
1.23E-01	8.92	2.67	hypothetical protein
1.05E-03	9721.32	2.67	hypothetical protein
4.76E-04	2491.13	2.63	Glutamate racemase
2.12E-02	122.59	2.63	Multidrug resistance protein MdtA
1.94E-02	441.22	2.55	Sec-independent protein translocase protein TatC
4.41E-04	7.45	2.49	hypothetical protein
9.62E-03	147.88	2.43	Ammonia channel
3.70E-03	279.88	2.43	hypothetical protein
7.90E-05	287.10	2.40	Flagella basal body P-ring formation protein FlgA
7.94E-03	273.44	2.40	Transposase
5.31E-03	59.46	2.39	Protein CyaE
9.51E-03	1627.58	2.34	hypothetical protein
2.69E-04	809.73	2.32	hypothetical protein
6.98E-03	141.62	2.32	HlyD family secretion protein
8.44E-02	7.47	2.31	Hydrolase in pqqF 5'region

Supplementary Table S10: Top 50 downregulated genes after growth on thiosulfate at pH 2.5 as compared to elemental sulfur pH 1.5.

p-value	FPKM	LOG2	Genes
3.62E-01	7.22	-5.42	hypothetical protein
2.77E-02	30.77	-5.24	hypothetical protein
3.47E-01	0.25	-5.24	hypothetical protein
3.35E-01	0.18	-5.14	hypothetical protein
3.76E-01	444.80	-5.00	hypothetical protein
1.06E-02	104.24	-5.00	hypothetical protein
4.63E-02	53.59	-4.83	hypothetical protein
2.45E-02	22.15	-4.73	hypothetical protein
2.24E-02	37.28	-4.66	hypothetical protein
3.36E-01	0.15	-4.61	hypothetical protein
3.77E-01	143.15	-4.59	hypothetical protein
3.78E-01	8.20	-4.55	hypothetical protein
3.20E-02	57.62	-4.49	hypothetical protein
3.95E-01	40.05	-4.40	tRNA-Asn(gtt)
3.82E-01	214.90	-4.39	hypothetical protein
3.82E-01	492.89	-4.34	hypothetical protein
2.58E-02	20.93	-4.32	Nodulation protein NolG
3.77E-01	27.82	-4.28	hypothetical protein
2.54E-02	111.48	-4.28	hypothetical protein
2.14E-02	316.48	-4.25	hypothetical protein
3.84E-01	237.89	-4.24	Single-stranded DNA-binding protein
4.75E-02	25.69	-4.23	hypothetical protein
2.06E-02	130.27	-4.18	hypothetical protein
3.81E-01	8.74	-4.16	DNA ligase
3.86E-01	115.20	-4.16	hypothetical protein
3.71E-01	16.67	-4.16	Helicase associated domain protein
3.80E-01	23.98	-4.15	hypothetical protein
3.84E-01	222.36	-4.13	hypothetical protein
3.84E-01	165.00	-4.13	hypothetical protein
3.80E-01	582.56	-4.11	hypothetical protein
3.87E-01	348.92	-4.11	hypothetical protein
3.85E-01	163.94	-4.11	hypothetical protein
3.87E-01	198.71	-4.10	hypothetical protein
3.82E-01	146.45	-4.10	hypothetical protein
3.86E-01	122.17	-4.10	hypothetical protein
3.86E-01	157.14	-4.09	hypothetical protein
3.83E-01	163.38	-4.09	hypothetical protein
3.84E-01	151.43	-4.08	hypothetical protein
3.87E-01	170.41	-4.08	hypothetical protein
3.87E-01	293.93	-4.07	hypothetical protein
3.84E-01	144.87	-4.05	hypothetical protein
3.87E-01	283.24	-4.05	hypothetical protein
3.80E-01	10.97	-4.05	hypothetical protein
3.87E-01	85.79	-4.04	hypothetical protein
3.86E-01	211.21	-4.03	hypothetical protein
3.85E-01	712.51	-4.01	hypothetical protein
3.86E-01	128.00	-4.00	hypothetical protein
3.88E-01	411.17	-3.99	hypothetical protein
3.73E-01	17.79	-3.98	hypothetical protein
3.81E-01	111.16	-3.95	hypothetical protein

Supplementary Table S11: Mean values for all sulfur species where concentrations for all S are in mM in mol of S.

S^0 Treatment

Day	pH	S^{2-}	SO_4^{2-}	S^0	$S_2O_3^{2-}$	SO_3^{2-}	Other SOI	Total SOI	Total Aqueous S	Total S
0	4.5	0.00	3.55	311.79	0.02	0.00	0.08	311.89	3.65	315.44
1	3.3	0.00	4.06	310.74	0.06	0.00	0.58	311.38	4.70	315.44
2	2.2	0.00	6.42	302.36	0.16	0.00	6.50	309.02	13.08	315.44
3	1.7	0.00	10.16	290.37	0.02	0.00	14.89	305.28	25.07	315.44
4	1.65	0.00	15.22	287.06	0.19	0.00	12.97	300.22	28.38	315.44
5	1.5	0.00	17.18	272.40	0.50	0.0004	25.35	298.26	43.04	315.44

$S_2O_3^{2-}$ Treatment

Day	pH	S^{2-}	SO_4^{2-}	S^0	$S_2O_3^{2-}$	SO_3^{2-}	Other SOI	Total SOI	Total Aqueous S	Total S
0	4.55	0.03	4.27	0.19	28.84	0.12	7.05	36.20	40.30	40.50
2	2.3	0.07	12.14	1.89	10.28	0.004	16.12	28.29	38.61	40.50
4	2.45	0.03	12.07	8.71	5.50	0.22	13.97	28.40	31.78	40.50

Supplementary Table S12: Values for S^0 in mM, for all methods used. **N.B.** the low values of HPLC S_T in the S^0 treatment from day 0-3, are most likely due to the nature of S^0 in the media where initially it is clumped and mostly out of solution. Variation in values for HPLC vs ($\sum S_{in\ media}$ - $\sum S_{aq}$) may also be attributed to external vs internal S^0.

S^0 Treatment

Day	pH	S^0 = HPLC S_{aq}	S^0 = HPLC S_T	S^0 = $\sum S_{in\ media}$ - $\sum S_{aq}$
0	4.5	0.00	10.37	311.79
1	3.3	0.00	63.33	310.74
2	2.2	0.00	128.28	302.36
3	1.7	0.00	219.28	290.37
4	1.65	0.00	261.29	287.06
5	1.5	0.00	172.11	272.40

$S_2O_3^{2-}$ Treatment

Day	pH	S^0 = HPLC S_{aq}	S^0 = HPLC S_T	S^0 = $\sum S_{in\ media}$ - $\sum S_{aq}$
0	4.55	0.00	0.19	0.19
2	2.3	0.00	1.45	1.89
4	2.45	0.00	2.36	8.71

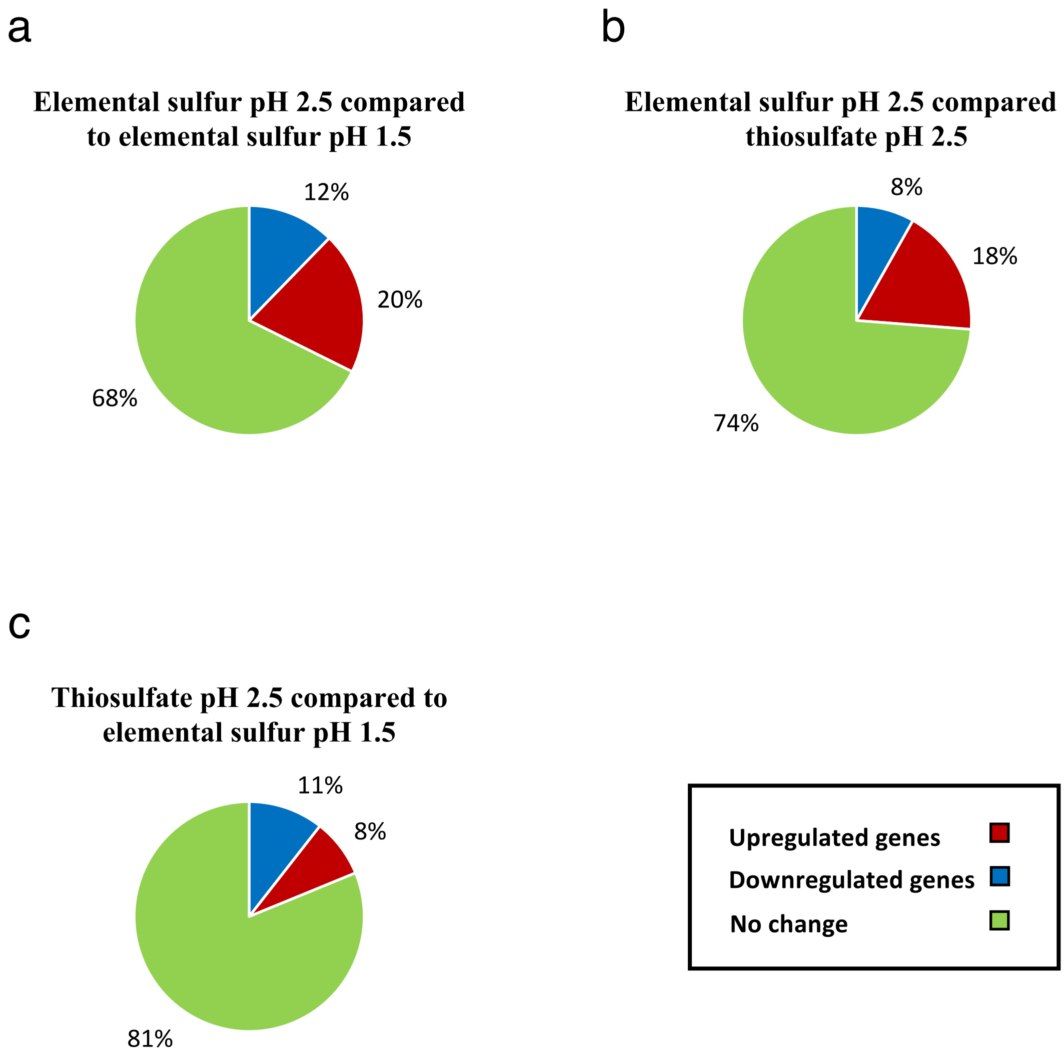

Supplementary Figure S1: Analysis of gene expression regulation on all genes after growth with S^0 or $S_2O_3^{2-}$ as energy source. Comparative gene expression for FPKM values based on Log2 ratio. (**a**) Growth on same substrate (S^0) at different points on pH and growth curve (pH 2.5 = day 3 / pH 1.5 = day 5), (**b**) growth to same pH (2.5) on different substrates and points on growth curve (S^0 = day 3 / $S_2O_3^{2-}$ = day 5), (**c**) growth until day 5 on different substrates and to different pH values ($S_2O_3^{2-}$ = pH 2.5 / S^0 = pH 1.5).

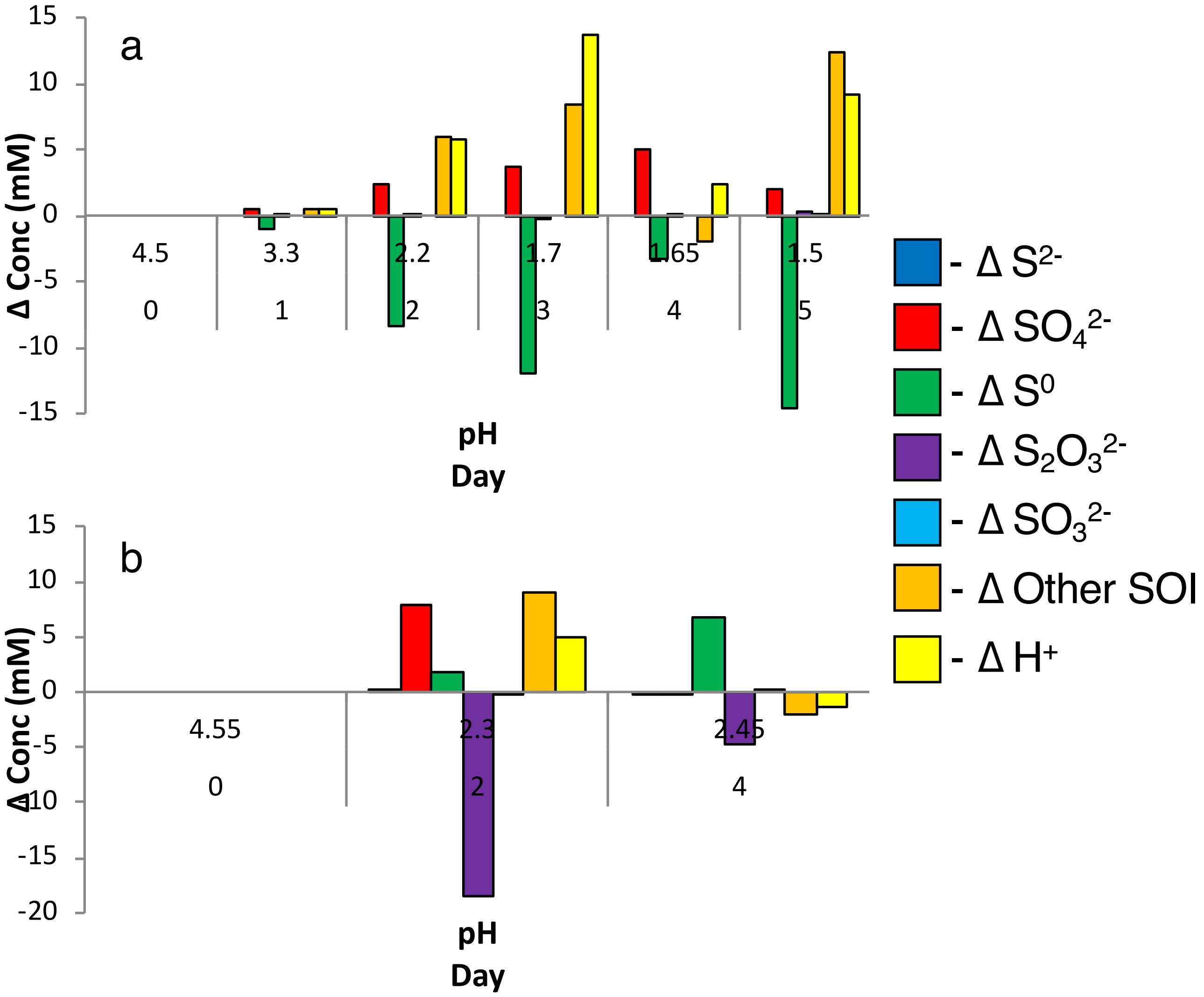

Supplementary Figure S2: Change in sulfur species and H^+ concentration per sampling point. (**a**) For S^0 media. (**b**) For $S_2O_3^{2-}$ media. Concentrations of all S species are mM in mol of S.

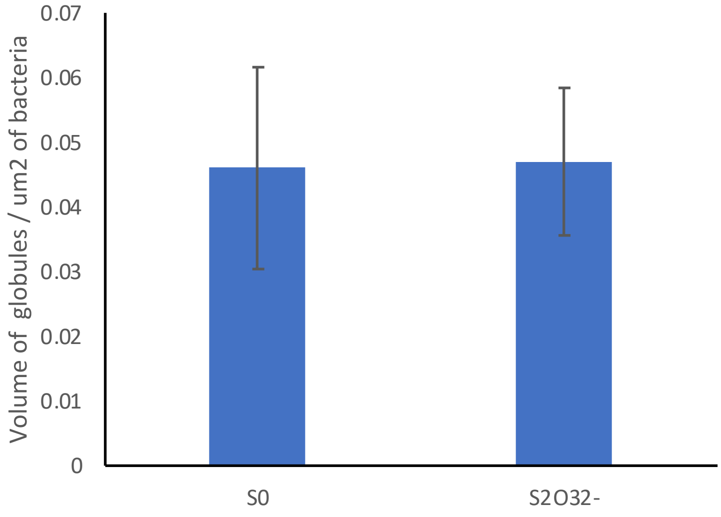

Supplementary Figure S3: Size in volume of the S^0 globules formed in *A. thiooxidans* cells when grown in either S^0 or $S_2O_3^{2-}$, analysis done via Image J.
