# Supplementary material for: New Insights Into Acidithiobacillus thiooxidans Sulfur Metabolism Through Coupled Gene Expression, Solution Chemistry, Microscopy, and Spectroscopy Analyses
Source: Front Microbiol. 2020 Mar 13;11:411. doi: 10.3389/fmicb.2020.00411 (PMC7082400; doi:10.3389/fmicb.2020.00411)
Supplement: Supplementary file 2 [file Data_Sheet_2.docx]

Supplementary Text and Analysis

Methods

*Metabolism Model Creation*

Stoichiometric mass balance based on net changes in solution sulfur and acid concentrations across time, within each treatment, were first used to identify the most likely sulfur reactions occurring (Supplementary Figure S2). These geochemical changes could be balanced via several possible sulfur reactions creating uncertainty in identification of the specific pathways being catalyzed (Supplemental Data). Then, we used the gene expression levels (Figure 3a) to refine the reaction pathways identified as most likely from our geochemical results assessing sulfur species and proton changes over time (Supplementary Figure S2). The resulting metabolic maps highlight the genes and pathways being potentially catalyzed (Figure 7). The best model predicting sulfur reactions was then determined by narrowing down the range of geochemically possible reactions through observed changes in gene expression levels, which provides clues as to which reactions are most likely occurring (Supplemental Data, Figure 7). Further sulfur metabolism models were developed for both S^0^ and S_2_O_3_^2-^ as initial sulfur sources, via stoichiometric balancing and the identified pathways from the entire suite of those proposed, observed, hypothesized and/or putatively suggested in the literature to be catalyzed by specific genes (Table 3; Figure 1), generating *A. thiooxidans* S^0^ and S_2_O_3_^2-^ dependent metabolism models (Supplemental Data and Figure 6).

Results

Stepwise reactions for Figure 6 follows.

**Step-Wise reactions**

6S^0^ → 2SO_4_^2-^ + 4S^Other SOI^ + 5H^+^ (Table 3, Eq 1)

37S_2_O_3_^2-^ + 0.5SO_3_^2-^ → 7S^0^ + 31.5SO_4_^2-^ + 36S^Other SOI^ + 20H^+^ (Table 3, Eq 7)

3S_2_O_3_^2-^ → 2S^0^ + 2SO_4_^2-^ + 2S^Other SOI^ + H^+^ (Table 3, Eq 8)

Figure 6a was theorized via mass balance of total experiment for ASOM-S^0^ i.e Eq (1) Table 3 to get the closest in describing S-species consumption/production and H^+^ production.

Step-wise reactions

4S^0^ + 4O_2_ + 4H_2_O → 4SO_3_^2-^ + 8H^+^ (1)

2SO_3_^2-^ + O_2_ → 2SO_4_^2-^ (2)

2S^0^ + 2SO_3_^2-^ → 2S_2_O_3_^2-^ (3)

2S_2_O_3_^2-^ + 0.5O_2_ + 2H^+^ → S_4_O_6_^2-^ + H_2_O (4)

Figure 6b was created using similar steps, based on the observed total S changes from day 0-2 for the ASOM-S_2_O_3_^2-^ test, Eq (7) Table 3.

Step-wise reactions

7S_2_O_3_^2-^ → 7S^0^ + 7SO_3_^2-^ (5)

7SO_3_^2-^ + 7H_2_O → 7SO_4_^2-^ + 14H^+^ (6)

0.5SO_3_^2-^ + O_2_ → 0.5SO_4_^2-^ (7)

30S_2_O_3_^2-^ + 7.5O_2_ + 30H^+^ → 15S_4_O_6_^2-^ + 15H_2_O (8)

6S_4_O_6_^2-^ + 21O_2_ + 18H_2_O → 24SO_4_^2-^ + 36H^+^ (9)

Figure 6c was also theorized the same as the others but based on ASOM- S_2_O_3_^2-^ total experiment (day 0-4) as in Eq (8) Table 3.

Step-wise reactions

3S_2_O_3_^2-^ + 0.75O_2_ + 3H^+^ → 1.5S_4_O_6_^2-^ + 1.5H_2_O (10)

S_4_O_6_^2-^ + H_2_O → S_3_O_3_^2-^ + SO_4_^2-^ + 2H^+^ (11)

S_3_O_3_^2-^ → 2S^0^ + SO_3_^2-^ (12)

S^0^ + SO_3_^2-^ → S_2_O_3_^2-^ (13)

S_2_O_3_^2-^ → S^0^ + SO_3_^2-^ (14)

SO_3_^2-^ + H_2_O → SO_4_^2-^ + 2H^+^ (15)

(Eq 13 + 14 can also be replaced directly to Eq 15)

Discussion

*RNA-seq on other metabolic characteristics*

Whereas we primarily focused on the genes involved in sulfur metabolism, RNA-seq analysis provides genome-wide quantitative information on gene expression patterns. This analysis revealed higher expression of a substantial number of genes of the flagellar assembly pathway at day 3 during growth on S^0^ as compared to day 5. The bacteria are probably more metabolically active in the exponential growth phase at day 3 and this could be linked to higher mobility. We also obtained evidence for differential expression of genes encoding ATP synthase components as well as genes encoding components of cytochrome C biogenesis, protein folding and membrane stability, providing additional insights into the distinct metabolic adaptations under the different growth conditions tested here.
